# Food-Web Complexity in Guaymas Basin Hydrothermal Vents and Cold Seeps

**DOI:** 10.1371/journal.pone.0162263

**Published:** 2016-09-28

**Authors:** Marie Portail, Karine Olu, Stanislas F. Dubois, Elva Escobar-Briones, Yves Gelinas, Lénaick Menot, Jozée Sarrazin

**Affiliations:** 1 Laboratoire Environnement Profond, REM/EEP, Institut Carnot Ifremer EDROME, Centre de Bretagne, Plouzané, France; 2 Laboratoire Ecologie Benthique, DYNECO, Ifremer, Centre de Bretagne, Plouzané, France; 3 Instituto de Ciencias del Mar y Limnología, Universidad Nacional Autónoma de México, Mexico City D.F., Mexico; 4 GEOTOP and Chemistry and Biochemistry Department, Concordia University, Montréal, Québec, Canada; Stockholm University, SWEDEN

## Abstract

In the Guaymas Basin, the presence of cold seeps and hydrothermal vents in close proximity, similar sedimentary settings and comparable depths offers a unique opportunity to assess and compare the functioning of these deep-sea chemosynthetic ecosystems. The food webs of five seep and four vent assemblages were studied using stable carbon and nitrogen isotope analyses. Although the two ecosystems shared similar potential basal sources, their food webs differed: seeps relied predominantly on methanotrophy and thiotrophy via the Calvin-Benson-Bassham (CBB) cycle and vents on petroleum-derived organic matter and thiotrophy via the CBB and reductive tricarboxylic acid (rTCA) cycles. In contrast to symbiotic species, the heterotrophic fauna exhibited high trophic flexibility among assemblages, suggesting weak trophic links to the metabolic diversity of chemosynthetic primary producers. At both ecosystems, food webs did not appear to be organised through predator-prey links but rather through weak trophic relationships among co-occurring species. Examples of trophic or spatial niche differentiation highlighted the importance of species-sorting processes within chemosynthetic ecosystems. Variability in food web structure, addressed through Bayesian metrics, revealed consistent trends across ecosystems. Food-web complexity significantly decreased with increasing methane concentrations, a common proxy for the intensity of seep and vent fluid fluxes. Although high fluid-fluxes have the potential to enhance primary productivity, they generate environmental constraints that may limit microbial diversity, colonisation of consumers and the structuring role of competitive interactions, leading to an overall reduction of food-web complexity and an increase in trophic redundancy. Heterogeneity provided by foundation species was identified as an additional structuring factor. According to their biological activities, foundation species may have the potential to partly release the competitive pressure within communities of low fluid-flux habitats. Finally, ecosystem functioning in vents and seeps was highly similar despite environmental differences (e.g. physico-chemistry, dominant basal sources) suggesting that ecological niches are not specifically linked to the nature of fluids. This comparison of seep and vent functioning in the Guaymas basin thus provides further supports to the hypothesis of continuity among deep-sea chemosynthetic ecosystems.

## Introduction

In the deep sea where organic inputs are limited, hydrothermal vents and cold seeps host original and luxuriant faunal communities that thrive on local chemosynthetic production [1]. Hydrothermal vents are characterised by the presence of high-temperature emissions that spring through cracks in the seafloor along oceanic ridges and back-arc basins. Cold seeps are characterised by fluids oozing out of the sediments along continental margins. In both ecosystems, fluids are enriched in reduced compounds that fuel microbial primary production. Bacteria and archaea use several electron donors (e.g. H_2_S, H_2_, CH_4_, Fe^2+^, Cu^2+^, Mn^2+^, NH_4_^+^) and electron acceptors (e.g.O_2_, NO_3_^-^, SO_4_^2-^) as energy sources to convert inorganic carbon into simple sugars [2]. The most common reduced chemicals are sulphide and methane, which are used by thiotrophic and methanotrophic microbes [3–5]. This microbial production sustains the development of dense faunal communities either as direct food sources or through symbiosis with invertebrates [6, 7]. In addition to chemosynthetic primary production, the degradation of endogenous or exogenous biomass and abiotic chemical processes produce dissolved organic matter (DOM) and particulate organic matter (POM) [8, 9]. DOM sustains free-living heterotrophic microbes and POM is used as a food source by benthic invertebrates [10]. Migrant organisms can also exploit the high productivity of these ecosystems and thereby contribute to the export of chemosynthetic organic matter to adjacent deep-sea ecosystems [11–13].

Within vent and seep ecosystems, faunal communities are heterogeneously distributed in a mosaic of assemblages defined by the presence of foundation species which are mainly symbiont-bearing invertebrates such as bivalves or tubeworms, or microbial mats formed by filamenteous bacteria. This patchy distribution is related to fluid emissions that govern the steep and unstable physico-chemical gradients at small spatial scales [14]. Fluid emissions provide the energy sources required by chemosynthetic producers, but generate toxic and highly fluctuating environmental conditions that limit faunal colonisation. Food webs along fluid-flux gradients may be structured through bottom-up (resource availability) and top-down (consumer pressure) controls [7]. In addition, the high faunal biomass, comparable to that of the most productive marine ecosystems [15], suggests that competitive interactions among taxa may structure the food webs (e.g. [11, 16]). Moreover, biotic interactions, through indirect effects and non-trophic relations, such as the engineering role of foundation species or facilitation by conspecifics, may further influence food-web dynamics [17, 18].

Vent and seep ecosystems exhibit numerous functional homologies and are characterised by phylogenetic similarities [19, 20]. Nevertheless, comparative studies at the community-scale show strong dissimilarities in faunal community structure between seeps and vents [14, 19, 21–25]. These dissimilarities may arise from dispersal barriers between these typically distant ecosystems (e.g. biogeographic barrier) and also, from specific ecological niches linked to the nature of fluids. The relative influence of these factors cannot be discerned in most existing seep and vent comparisons [25]. In the Guaymas Basin (Gulf of California, Mexico), seep and vent ecosystems are found in close proximity (ca. 60km), similar sedimentary settings and comparable depths (ca. 2000m). The absence of biogeographic barriers offers the opportunity to specifically address the influence of local environmental factors on faunal communities. Our recent study demonstrated similar patterns of macrofaunal diversity, density and taxonomic composition across these two ecosystems [26]. Hydrogen sulphide and methane concentrations along fluid flow gradients were major community structuring factors, with vent fluid specificities (e.g. temperature, metals) playing a minor role. Furthermore, the two ecosystems shared 85% of identified species providing evidences of faunal exchanges between the two ecosystems. All together, these results supported the hypothesis of continuity among chemosynthetic ecosystems [27] and raised questions about ecosystem functioning similarities.

This follow-up study aims to assess and compare Guaymas seep and vent ecosystem functioning through the analysis of food webs. Carbon and nitrogen stable isotope analyses are particularly adapted to the study of food webs in deep-sea remote habitats where direct observations and gut analyses are limited. δ^13^C and δ^15^N signatures, studied together, help identify the basal sources sustaining food webs, consumer trophic relationships and the presence of inter- and intraspecific trophic competition within communities [28]. At the community level, metrics can be extracted from the overall δ^13^C - δ^15^N isotopic space to address food-web complexity and estimate niche diversification at the base of the food web, the number of trophic levels, trophic diversity, specialisation and redundancy [29–31]. This study addresses the following specific questions: (1) Which biosynthetic pathways sustain vent and seep communities in the Guaymas Basin? (2) Do common species, shared by several assemblages, rely on specific basal sources? (3) What are the trophic relationships among species? (4) How does food-web structure vary according to environmental conditions? And finally, (5) are factors regulating the functioning common or specific to seep and vent ecosystems?

## Materials and Methods

### Study sites

**Fig 1:**
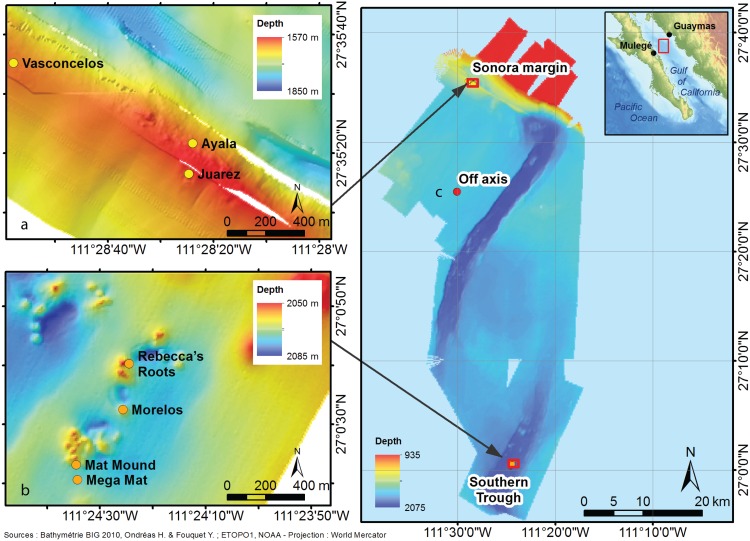
Location of the study sites in the Guaymas Basin. (a) cold seeps on the Sonora margin (Ayala, Vasconcelos and Juarez sites), (b) hydrothermal vents on the Southern Trough (Rebecca’s Roots, Mat Mound, Morelos and Mega Mat sites) and (c) the off-axis reference site located in between.

The Biodiversity and Interactions in the Guaymas Basin (BIG) cruise was held in 2010 on board the oceanographic research vessel *L'Atalante* equipped with the *Nautile* submersible. The Mexican Secretariat of Foreign Relations granted a work permit to carry out research in Mexican waters (DAPA/2/281009/3803, 28 October 2009). In this study, we focused on three areas in the Guaymas Basin located in the central portion of the Gulf of California (27°N, 111.5°W) (Fig 1): (1) cold seeps on the Sonora margin transform faults (27°36’N, 111°29’W) at 1550 m depth, (2) a large hydrothermal field on the Southern Trough depression (27°00’N, 111°24’W) at 1900 m depth and (3) an off-axis reference site (27°25’N, 111°30’W) located at 1500 m depth (G_Ref). The sampling design is detailed in ref. [26].

**Fig 2:**
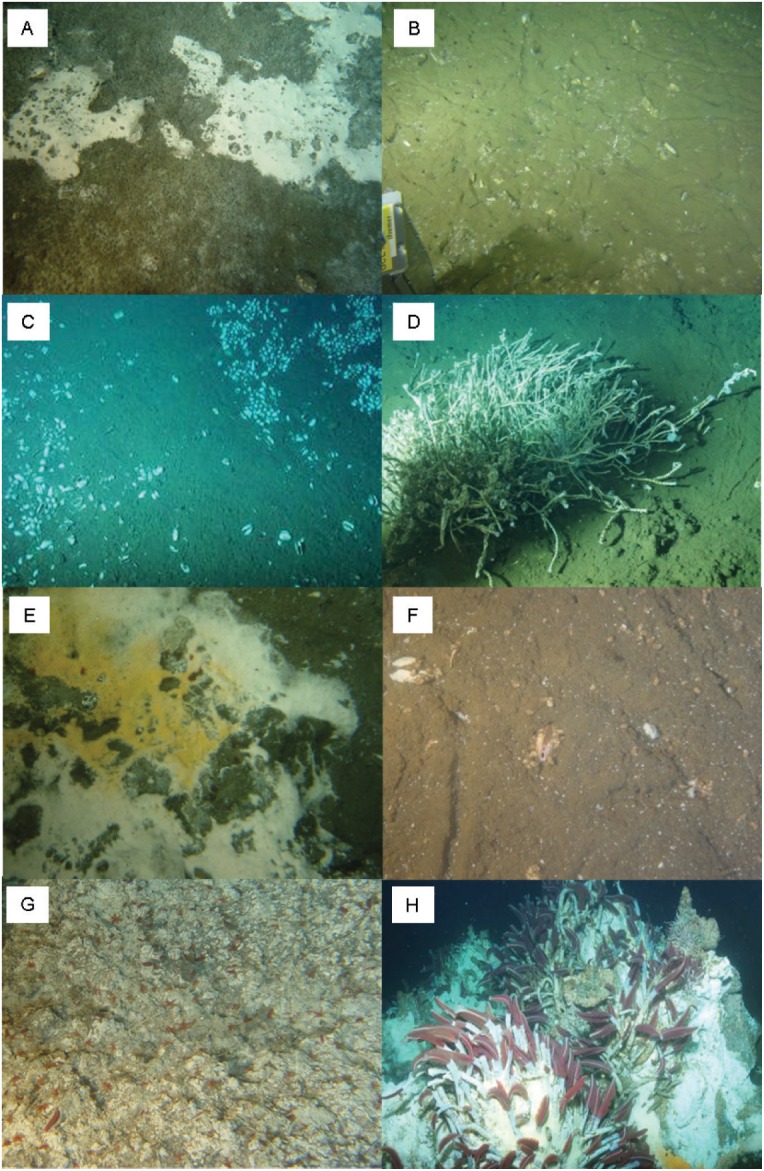
Images of the studied assemblages at seeps. (A) *Beggiatoa* spp. microbial mat (S_Mat) and *Hyalogyrina* sp. Gastropoda (S_Gast), (B) *Archivesica gigas* Vesicomyidae (S_VesA), (C) *Phreagena soyoae* Vesicomyidae (S_VesP), (D) *Escarpia spicata and Lamellibrachia barhami* Siboglinidae (S_Sib) and vents: (E) *Beggiatoa* spp. microbial mat (V_Mat), (F) *Archivesica gigas* Vesicomyidae (V_VesA), (G) *Paralvinella grasslei* and *P*. *bactericola* Alvinellidae (V_Alv) and (H) *Riftia pachyptila* Siboglinidae (V_Sib).

At the Sonora margin seeps (S_), we investigated food webs of five assemblages located in three different sites (Fig 2 and Table 1). At the Vasconcelos site, the S_Mat assemblage featured microbial mats dominated by the genus *Beggiatoa*; S_Gast was dominated by *Hyalogyrina* sp. gastropods; and S_VesA by *Archivesica gigas* vesicomyids. At the Ayala site, another vesicomyid assemblage S_VesP featured *Phreagena soyoae*. At the Juarez site, the S_Sib assemblage was dominated by two species of siboglinid tubeworms found on carbonate concretions, *Escarpia spicata* and *Lamellibrachia barhami*.

**Table 1:** Abbreviations and locations of the different assemblages studied in the Guaymas Basin.

Abbreviation	Foundation taxon	Substratum	Site	Ecosystem	Latitude	Longitude
G_ref	None	Soft	Off-axis	Background	27°25.483' N	111°30.076' W
S_Mat	*Beggiatoa* spp.	Soft	Vasconcelos	Seep	27°35.580' N	111°28.986' W
S_Gas	*Hyalogyrina* sp.	Soft	Vasconcelos	Seep	27°35.583' N	111°28.982' W
S_VesA	*A*. *gigas*	Soft	Vasconcelos	Seep	27°35.587' N	111°28.963' W
S_VesP	*P*. *soyoae*	Soft	Ayala	Seep	27°35.365' N	111°28.395' W
S_Sib	*E*. *spicata*, *L*. *barhami*	Hard	Juarez	Seep	27°35.274' N	111°28.406' W
V_Mat	*Beggiatoa* spp	Soft	Mega Mat	Vent	27°00.445' N	111°24.530' W
V_VesA	*A*. *gigas*	Soft	Morelos	Vent	27°00.547' N	111°24.424' W
V_Sib	*R*. *pachyptila*	Hard	Mat Mound	Vent	27°00.386' N	111°24.576' W
V_Alv	*P*. *grasslei*, *P*. *bactericola*	Hard	Rebecca’s Roots	Vent	27°00.664' N	111°24.412' W

At the Southern Trough vent (V_), we investigated food webs of four assemblages (Fig 2 and Table 1). At the Mega Mat site, the V_Mat assemblage featured white *Beggiatoa* spp. microbial mats. At the Morelos site, the V_VesA assemblage was dominated by *A*. *gigas* vesicomyids. At the Mat Mound site, the V_Sib assemblage was dominated by the siboglinid tube worm *Riftia pachyptila* established on sulphide substratum. Finally, at the Rebecca’s Roots sulphide edifice, the V_Alv assemblage was characterised by two alvinellid polychaetes, *Paralvinella grasslei* and *P*. *bactericola*. Except for the periphery of S_Sib, which was not covered in this study due to the absence of faunal collections for stable isotopic analyses, the assemblages herein were similar to those in our macrofaunal community structure study [26].

The physico-chemical characterisation of these assemblages was detailed in ref. [26]. Guaymas seep and vent assemblages belonged to different habitat groups according to the concentration of reduced compounds (i.e. methane and hydrogen sulphide) discriminating high fluid-flux assemblages (seeps: S_Gast and S_Mat, vents: V_Sib, V_Alv and V_Mat) from low fluid-flux assemblages (seeps: S_Sib, S_VesA and S_VesP, vents: V_VesA) regardless ecosystems. Low fluid-flux assemblages were characterized by low concentrations of methane (from 1 to 50 μM) and sulphide (from undetected to 1700 μM) while high fluid-flux assemblages were characterized by high concentrations of methane (from 300 to 900 μM) and sulphide (from 9000 to 31000 μM). Among common assemblages across ecosystems, microbial mat and vesicomyid assemblages belonged to the same respective habitat groups whereas siboglinid habitats differed strongly, with S_Sib characterised by lower fluid input than V_Sib.

In addition to reduced compounds, at vents, specific inputs of hydrothermal fluids showed temperature anomalies (from 7°C to 56°C), low pH, and high manganese and ammonium concentrations.

Methane and temperature were the only two variables available from all habitats to test for the influence of environmental conditions on ecosystem functioning. Methane was used as a common proxy of seep and vent fluid-fluxes and temperature as a vent-specific proxy.

### Sample processing for stable isotope analyses

#### Faunal samples

Faunal samples used for stable isotope analyses were extracted from the quantitative assemblage samples [26] and supplemented with non-quantitative faunal samples (net and suction sampler). Isotope analyses were carried out on macrofaunal taxa accounting for at least 90% of the total abundance per assemblage. Some meiofaunal taxa (Copepoda and Nematoda) were added when found in sufficient abundance. In total, 501 specimens belonging to 70 taxa were analysed.

Preservation- On board, the largest, most dominant mega- and macrofaunal species were sorted and frozen (-80°C) and/or preserved in 70% alcohol (A). The remaining organisms were sieved on a stack of four sieves of decreasing mesh size (1, 0.5, 0.3 and 0.25 mm), fixed in 4% buffered formaldehyde for 24 h, rinsed and finally preserved in 70% alcohol (FA). In the laboratory, all faunal samples were sorted and identified to the lowest taxonomic level possible. Phloxine B was added for 1 h to small fractions (0.3 and 0.25 mm) then rinsed to improve sorting (FAP). All frozen preserved samples were selected for isotopic analyses and A-, FA- or FAP-preserved samples were additionally analysed to characterise the overall food webs.

Sample preparations- In the laboratory, all organisms were first rinsed in distilled Milli-Q water. For large specimens, muscle tissue was selected; for intermediate-sized specimens, gut content was removed; and for small specimens, the whole body was used for analysis and, in some instances, several specimens were pooled to reach the minimum required weight. Samples were freeze-dried and ground into a homogeneous powder using a ball mill. Tissue was precisely weighed (ca. 1 mg) in tin capsules for carbon and nitrogen isotope analyses. For samples containing carbonates (Gastropoda, small Bivalvia, Aplacophora, Actinaria, Ophiuridae, Copepoda and Amphipoda), a subset was acidified to remove inorganic carbon and measure the δ^13^C signature of organic carbon only. Acidification was carried out by the addition of 0.1 M HCl, drop by drop, until effervescence ceased. The sample was then dried at 60°C under a fume extractor to evaporate the acid. To prevent the loss of dissolved organic matter, samples were not rinsed [32, 33].

#### Sediment samples

On board, sediment cores were sliced in 2-cm thick sections and frozen (-80°C). Back in the laboratory, each section was freeze-dried and gently ground with a mortar and pestle and sieved on a 100-μm mesh to remove large detritus. Then, samples were ground using a ball mill. Subsamples of sediment sections were pooled and re-homogenised to characterise three sections: 0–2, 2–6 and 6–10 cm. For each section, 10 mg of sediments were weighed in tin capsules for δ^15^N. The δ^13^C signature of organic carbon was obtained as described above for specimens containing carbonates, with a 1 M HCl solution.

### Bulk stable isotope analyses

Each faunal and sediment sample was analysed on a Eurovector elemental analyser coupled to an Isoprime stable isotope ratio mass spectrometer (EA-IRMS, GV Instruments, now Elementar Americas Inc.). Three certified (IAEA-CH-6 Sucrose, δ^13^C = -10.45 ± 0.03‰; IAEA-N-1-ammonium sulphate, δ^15^N = -0.43 ± 0.05‰) and laboratory standards (β-alanine from Sigma-Aldrich, standardised in-house against several certified materials, δ^13^C = -26.08 ± 0.22‰, δ^15^N = -2.24 ± 0.17‰) were inserted between series of six to eight samples. Analytical precision based on the standard deviation of replicates of internal standards was ≤ 0.2‰ for both δ^13^C and δ^15^N. All values are expressed in δ (‰) notation with respect to VPDB (δ^13^C) and air (δ^15^N):

$$\delta \text{X}\left(‰\right)=[({\text{R}}_{\text{sample}}/{\text{R}}_{\text{standard}})-1]\text{x}10^{3},$$

where X is either ^13^C or ^15^N, R_sample_ is the ^13^C/^12^C or ^15^N/^14^N isotope ratio in the sample and R_standard_ is the ^13^C/^12^C or ^15^N/^14^N isotope ratio for the VPDB standard (δ^13^C) or air (δ^15^N).

### Methane carbon isotope analyses

The stable isotope composition of methane sampled above faunal assemblages with the CALMAR benthic chamber [34] was measured by ISOLAB b.v. (Neerijnen, The Netherlands) using a MAT Finnigan delta S mass spectrometer (San Jose, CA, USA) coupled to a gas chromatograph by a GC/C II interface.

### Data analyses

#### Preservation effects on stable isotope ratios

According to the literature, formaldehyde and alcohol preservation methods may or may not significantly bias the δ^13^C and δ^15^N signatures [35–41]. Phloxine B has also been shown to induce a potential bias depending on the species [42]. However, biases are usually limited compared to the natural variability in marine food sources (shifts in δ^13^C values ~1.1‰, δ^15^N values ~0.5‰). We nonetheless tested the potential biases introduced by the preservation methods on our dataset using a two-way unbalanced and non-parametric analysis of variance [43], with preservation treatments and species as factors. The species effect was always significant whereas alcohol (9 species, 65 individuals), formaldehyde (11 species, 84 individuals) or phloxine B (7 species, 47 individuals) effects on δ^13^C and δ^15^N signatures were not significant (p > 0.05). The interaction terms were never significant. Therefore, we did not apply any correction factor.

#### Faunal trophic guilds

Trophic guilds were classified into symbiont hosts, bacterivores/archivores, detritivores/scavengers, commensals/parasites and predators, with the bacterivorous/archivorous trophic guild referring to deposit feeders specialised in the consumption of microbes ([36], S1 Table). When available, trophic guilds from the literature were assigned to species. For species with unknown diets, trophic guild assignment was determined within each assemblage based on trophic guilds identified from other species within the same family along with the comparison of their stable isotope ratios with other species with well-defined trophic guilds. Endosymbiotic species within seep and vent ecosystems are well known. Bacterivorous/archivorous taxa usually feature depleted δ^15^N signatures, close to those of endosymbiotic species [44]. Discrimination of predators from detritivores/scavengers based on δ^15^N signatures is not always efficient [44]. However, since mega- and macrofaunal communities were exhaustively characterised within this study, species that belong to families with known predator species were classified as predators only when potential prey were identified. Prey were identified based on an enrichment (from consumer to its prey) of 3.4‰ for δ^15^N and 1‰ for δ^13^C [45], taking into account the intraspecific isotope variability. Nonetheless, since only large meiofaunal taxa were studied, predator and prey numbers may be underestimated.

#### Basal source contributions

Potential dominant basal sources at both Guaymas seeps and vents were similar, including photosynthesis-derived OM, endogenous methanotrophic and thiotrophic primary producers as well as chemo-organotrophic microbes relying on hydrocarbons higher than methane [46]. Emissions of these higher hydrocarbons occur at the substratum interface in Guaymas chemosynthetic ecosystems [47, 48] due to the degradation of diatomaceous, organic-rich sediments enhanced by steep temperature gradients in the sediments overlying the young ocean crust [49–51]. At vents, the thermocatalytic percolation of OM is further enhanced by the ascent of hot hydrothermal fluids [52].

At seeps and vents, primary producers have distinct δ^13^C signatures (Table 2) and their contributions in food webs can be monitored because consumers usually show low δ^13^C enrichment (ca. 1‰) relative to their food source [53]. Thiotrophic producers in both ecosystems have been shown to mainly utilise either the Calvin-Benson-Bassham cycle (CBB) or the reductive tricarboxylic acid cycle (rTCA) [54–56]. Each cycle has distinct isotopic signals, with the rTCA cycle leading to enriched δ^13^C signatures compared with the CBB cycle. In our study, vesicomyid and solemyid bivalve species, known to rely on their endosymbiotic thiotrophic bacteria that use the CBB cycle [57–59], were used to constrain the δ^13^C range of CBB thiotrophy in both seeps and vents. Because siboglinid polychaetes rely on thiotrophic symbionts fixing carbon via both cycles [54], they cannot be used as proxies for specific thiotrophic metabolisms. Methanotrophic producers usually assimilate methane with little or no carbon-isotope fractionation and are thus defined by the methane δ^13^C value [60–62]. In the Guaymas Basin, methane δ^13^C signatures are known to vary both within and between ecosystems [63–65]. Variations across ecosystems were further supported by seawater measurements taken above the studied assemblages showing a shift of methane δ^13^C from -41.8 ± 0.8‰ at vents to -52.3 ± 1.2‰ at seeps (S1 Table). Within assemblages and especially soft-sediment ones, methane δ^13^C can vary according to biogeochemical processes leading to depleted values in deep layers. Interestingly, extremely depleted values have been reported for archivorous sub-surface consumers feeding specifically on methanotrophic archaea involved in microbial consortia responsible for the anaerobic oxidation of methane (AOM) [66]. Finally, stable isotope ratios of petroleum have a low carbon fractionation factor (0.5‰) even through evaporation, microbial decomposition, or physical weathering processes [67–69]. Petroleum δ^13^C signatures are thus used as proxy of chemo-organotrophic microbes relying on petroleum.

**Table 2:** Isotope signature estimates for the potentially dominant basal sources in seeps and vents in the Guaymas Basin.

Basal source	Ecosystem	δ^13^C (‰)	δ^15^N (‰)	Reference
Photosynthetic-POM	Seep, Vent	-19.6 ± 0.7‰	9.2 ± 1.2‰	[65, 70]
Thiotrophy (CBB)	Seep, Vent	-35 to -30‰	-	[55], this study
Thiotrophy (rTCA)	Seep, Vent	-15 to -10‰	-	[55]
Methanotrophy	Seep	-60 to -50‰	-	[65], this study
Methanotrophy	Vent	-50 to -40‰	-	[63, 64], this study
Petroleum	Seep	-21 to -20‰	-	[71]
Petroleum	Vent	-23 to -21‰	-	[71]

Although δ^15^N signatures typically do not differentiate primary producers, they can vary according to their allochthonous or autochthonous origins. In the Guaymas Basin, photosynthesis-derived matter degraded in the water column is enriched in δ^15^N (Table 2). While lacking the δ^15^N of chemosynthetic producers, these are usually associated with low or even negative values, characteristic of local inorganic nitrogen sources (NO_3_^-^, NH_4_^+^, N_2_) [45, 62, 72].

Although stable isotope mixing models are increasingly used to quantify consumer diets, their use was not possible in our study owing to the presence of a large number of potential sources and the lack of δ^15^N values for chemosynthetic producers. In addition, although sulphur isotope composition might have helped to further discriminates carbon sources and quantifies species diets [73], the quantity of tissue for most macrofaunal specimens did not allow the analysis of both C/N and S isotopic ratios. Therefore, only trends of the predominant basal sources within assemblages are discussed.

#### δ^15^N baseline

For comparison across ecosystems, an estimate of a δ^15^N baseline is necessary to define the absolute trophic position of an organism [74]. Even though δ^15^N signatures of sources were not measured here, they are expected to vary within and between chemosynthetic ecosystems of the Guaymas Basin. To assess habitat variability in δ^15^N baselines, regressions were tested between the mean δ^15^N signatures of primary consumers specialising in microbial primary producers (endosymbiotic species and bacterivores) and the mean δ^15^N signatures of the other consumers. Because these variations in δ^15^N baselines may be driven by the availability of nitrogen sources, we further tested the correlation between mean faunal δ^15^N signatures and NH_4_^+^ concentrations that were available for soft-sediment assemblages [26]. All mean comparisons of isotope ratios were carried out using the non-parametric Kruskal-Wallis test, followed by a LSD rank test for pairwise comparisons [75].

#### Food-web metrics

Stable isotope analyses have been primarily used to study trophic diets. However, they can also reflect habitat characteristics, thus representing two crucial factors that define an organism’s ecological niche [76]: trophic and habitat compartments. The δ^13^C and δ^15^N signatures of basal sources are associated with a potential degree of variability among and within habitats according to local biogeochemical processes [77–81]. In analogy to Grinnell and ultimately Hutchinson, an ecological niche can be represented as an n-dimensional hyper volume partitioned into scenopoetic axes (habitat components) and bionomic axes (trophic components) [82, 83]. The δ^13^C - δ^15^N space represents species’ “isotopic niche” [76] and thus potentially illustrates the realised trophic niche [84, 85].

In this study, food-web structures at the community level were described using Layman community-wide metrics through a Bayesian approach [29, 30]. To compare these indices among assemblages, the δ^13^C signature ranges of possible basal sources must be similar [29], which is a valid hypothesis here. The Bayesian approach allows for the propagation of sampling error related to the mean estimations of stable isotope ratios for community components [30]. This calculation returns a posterior distribution of metric estimates providing a measure of uncertainty and allowing statistical comparisons. Bayesian metrics also have the advantage of being less affected by variation in the number of community components, thus allowing comparisons among communities [30].

Food web metrics used in our study are summarized in Table 3. Extents of isotope niches can be addressed by the small sample-size-corrected standard ellipse areas (SEAc; expressed in ‰^2^) and the Bayesian standard ellipse area (SEAb). Niche diversification at the base of food webs can be estimated by the δ^13^C signature range (CR) and trophic lengths by the δ^15^N signature range (NR). The average trophic specialisation within a community can be measured by the mean distance to the centroid (CD) that corresponds to the average Euclidian distance of each species component to the centroid. The assessment of trophic redundancy within a community has been recently proposed using a composite metric (coefficient of variation of the nearest neighbour distance, CVNND) [86]. This metric is equal to the ratio of the standard deviation to the mean of nearest neighbour distance representing the density of species packing, with low values suggesting increased trophic redundancy. Metrics were calculated and compared using the siber.hull.metrics function from the Stable Isotope Analysis in R package (SIAR; [87, 88] in R software). SEAs were calculated and compared using the SIAR and Stable Isotope Bayesian Ellipses packages (SIBER; [30] in R).

**Table 3:** Resume of the food web metrics with their acronyms and significance.

Acronym	Metric	Significance
SEAs	Standard ellipse areas	Overall extent of food web
CR	Carbon range	Basal niche diversification
NR	Nitrogen range	Trophic lengths
CD	Mean distance to the centroid	Average trophic specialisation
CVNND	Coefficient of variation of the nearest neighbour distance	Trophic redundancy

To assess the variations in food-web complexity along fluid-flux gradients, parametric regression models between food-web metrics and either methane concentrations, used as a proxy for both seep and vent fluid fluxes, or temperature used as a vent-specific proxy, were tested. Furthermore, the relationship between functioning and diversity was explored by constructing parametric regression models between food-web Bayesian metrics and macrofaunal alpha diversity [26].

## Results

### Community-wide food web description

δ^13^C and δ^15^N mean values of sources and consumers together with trophic guilds are listed in S1 Table.

#### Dominant basal sources

**Fig 3:**
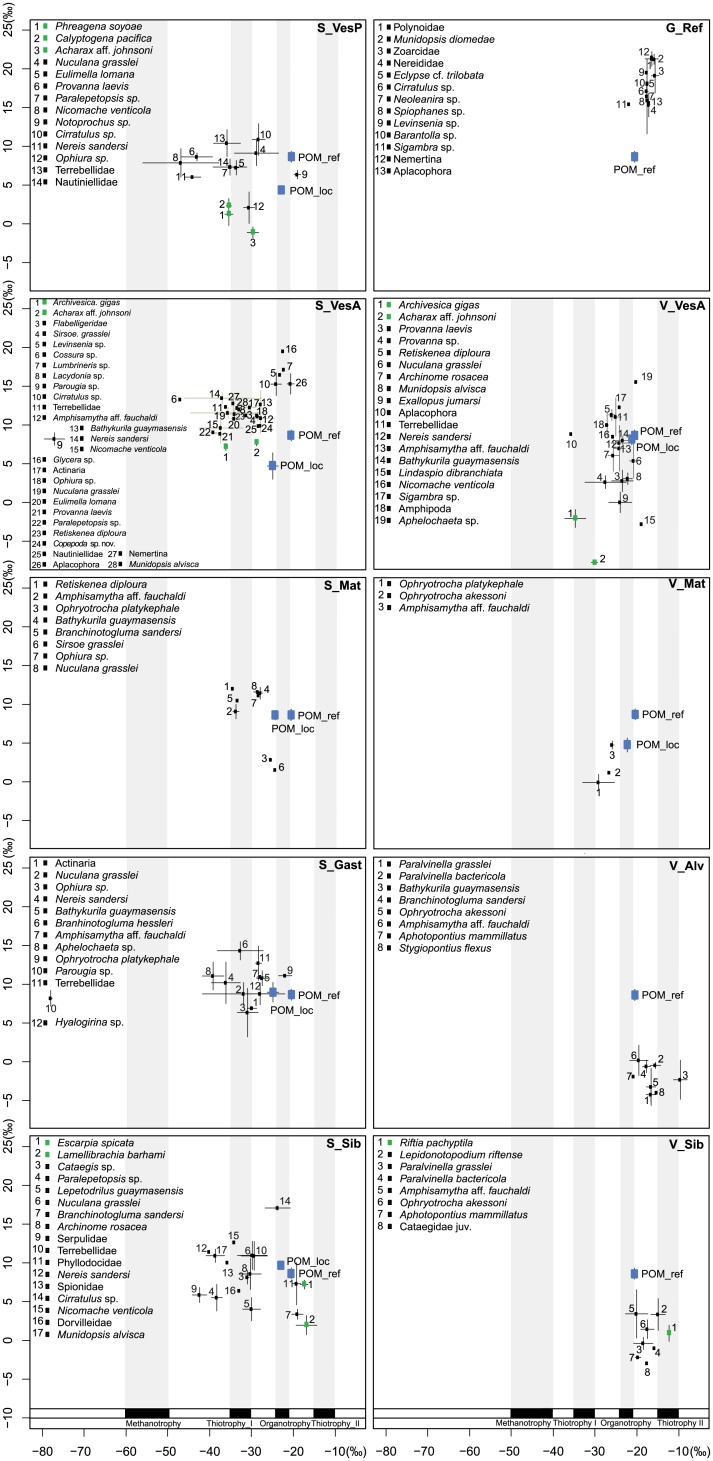
Biplots of carbon (δ^13^C) and nitrogen (δ^15^N) mean ± SD signatures of consumers and food resources. Endosymbiotic species are figured in green and heterotrophic consumers in black symbols. Isotopic signatures of the reference site POM (POM_ref) are provided as an estimator of the photosynthesis-derived organic matter, the values of POM within each assemblage (POM_loc) are used to characterise the local mixture between endogenous and exogenous organic matter. δ^13^C ranges of endogenous microbes are represented while their δ^15^N signatures are unknown. Thiotrophy I and II refer to the CBB and rTCA cycles, respectively.

At G_Ref, all taxa showed δ^13^C signatures close to that of POM and δ^15^N signatures were enriched by at least 6‰ and up to 12‰ compared with that of POM (Fig 3). Isotope ratios of particulate organic matter sampled within seep and vent soft-sediment assemblages (POM_loc), reflecting the local mixture between exogenous (photosynthesis-derived, POM_ref) and endogenous (chemosynthesis-derived) organic matter, attested to the high contribution of the former one to the POM pool. However, at seep and vent assemblages, photosynthetic POM was not considered a dominant source with the exception of a few taxa: *Notoproctus* sp. at S_VesP; *Glycera* sp., *Lumbrineris* sp., *Levinsenia* sp., *Cirratulus* sp. and Aplacophora at S_VesA; *Cirratulus* sp. at S_Sib; and *Aphelochaeta* sp. at V_VesA. All other taxa had distinct δ^13^C signatures and/or depleted δ^15^N values in comparison with photosynthetic POM, suggesting a relatively low contribution of this source.

At the community level, despite similar potential basal sources, the dominant sources within food webs differed, especially among vents and seeps. All seep assemblages appeared predominantly sustained by CBB-cycle thiotrophic and methanotrophic producers. At S_Gast and S_VesA, the highly depleted δ^13^C ratio of *Parougia* sp. reflected a specialised archivorous diet based on the AOM consortium. Thiotrophy using the rTCA cycle appeared to be the dominant basal source for only four taxa in the S_Sib assemblage including the siboglinid species *E*. *spicata* and *L*. *barhami*, together with a phyllodocid and *Branchinotogluma sandersi*. At vents, V_Mat and V_VesA assemblages appeared to mainly rely on petroleum-derived OM and thiotrophy using the CBB cycle whereas dominant sources at V_Sib and V_Alv were related to petroleum-derived OM and thiotrophic producers using the rTCA cycle.

#### Trophic guilds

**Fig 4:**
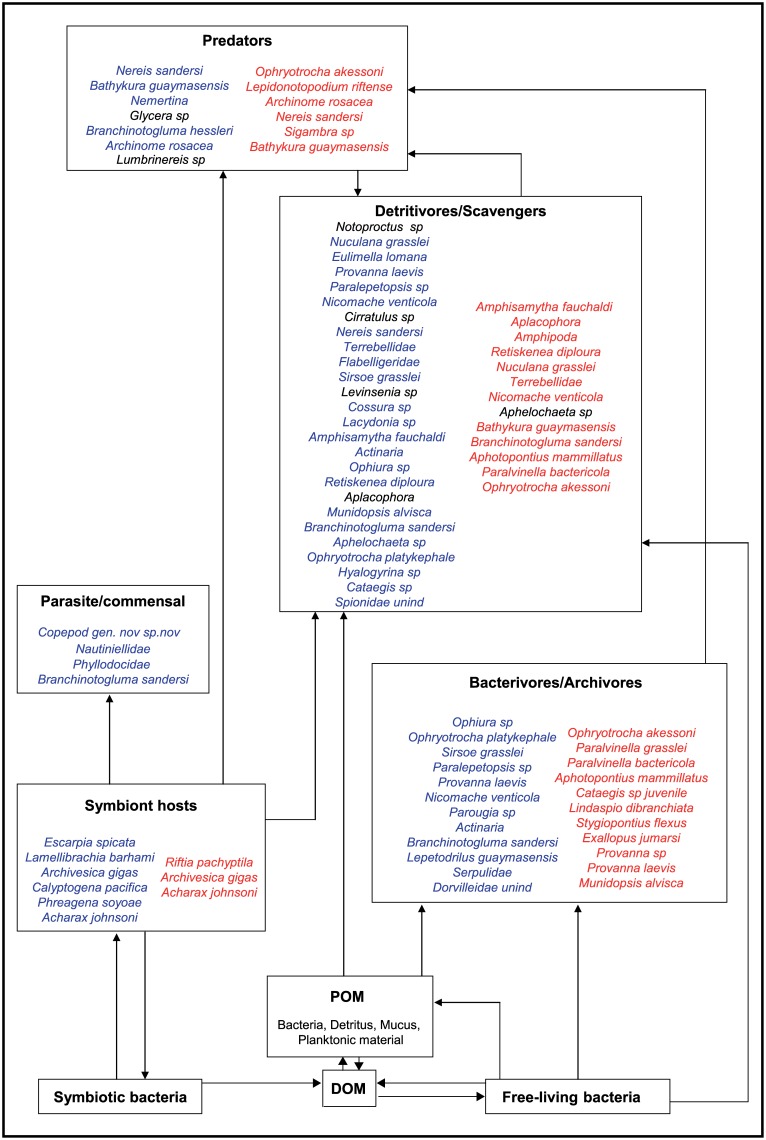
Trophic guilds of Guaymas seeps and vents taxa. Species from seeps and vents are figured in blue and red, respectively, except those primarily sustained by photosynthetic organic matter that are shown in black symbols.

**Fig 5:**
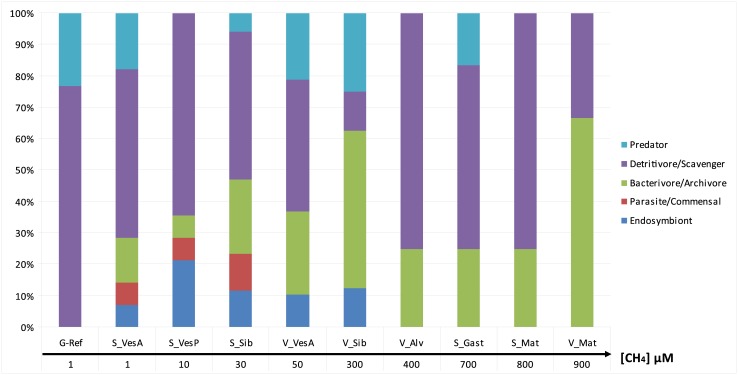
Relative number of taxa per trophic guilds ordered along an increasing fluid-flux gradient with methane concentrations used as a proxy.

At G_Ref, the community was composed of detritivores/scavengers and few predators (S1 Table). The trophic guilds at seep and vent ecosystems were more diverse including endosymbiotic species, bacterivorous/archivorous specialists, detritivores and predators (S1 Table and Fig 4). Commensal/parasitic species were only found at seeps. Some species were related to distinct trophic guilds depending on where the assemblages were found. Variability in the relative proportion of trophic guilds did not show any clear patterns with regard to fluid flux or ecosystem type, with the exception of endosymbiotic species and their potential parasites that were absent from most high fluid-flux assemblages at both seeps and vents (Fig 5).

#### Biotic interactions

Commensal/parasitic relationships- At seep vesicomyid assemblages, nautiniellid polychaetes had similar δ^13^C values and enriched δ^15^N signatures, from 2 to 6‰, in comparison with their bivalve hosts, suggesting a parasitic relationship (Table 4). In addition, copepods belonging to a new genus/new species (description in progress, Ivanenko unpublished data) were sampled from the gill lamellae of *A*. aff. *johnsoni* (S1 Fig). Their isotope ratios suggested potential parasitic relationships due to their relatively similar δ^13^C signatures and 2‰ ^15^N enrichment compared with *A*. aff. *johnsoni*. At seep siboglinid assemblage, *B*. *sandersi* and phyllodocid polychaetes appeared to be closely associated with the endosymbiotic tube worms, *E*. *spicata* and *L*. *barhami*, respectively, suggesting a potential commensal/parasitic interaction. No direct predation was suspected because their δ^13^C signatures were depleted and their δ^15^N signatures were relatively similar to the endosymbiotic species.

**Table 4:** Biotic interactions identified at the different study sites.

**Commensal/parasitic relationships**		
Site	Potential parasite	Host
S_VesP, S_VesA	Nautiniellid polychaetes	*C*. *pacifica* vesicomyids, *A*. aff. *johnsoni* solemyids
S_VesA	Copepods	*A*. aff. *johnsoni* solemyids
S_Sib	*B*. *sandersi* polychaetes	*E*. *spicata* siboglinids
	Phyllodocid polychaetes	*L*. *barhami* siboglinids
**Predator/prey relationships**		
Site	Predator	Potential prey
G_ref	Polynoidae	*Cirratulus* sp., *Neoleanira* sp., *Eclypse cf*. *trilobata*, *Barantolla* sp
	Nemertina	*Cirratulus* sp., *Neoleanira* sp., *Eclypse cf*. *trilobata*, *Barantolla* sp
	Zoarcidae	*Neoleanira* sp., *Spiophanes* sp., Aplacophora, Nereididae undet
S_VesA	*B*. *guaymasensis*	*Ophiura* sp., *Sirsoe grasslei*, *A*. aff. *johnsoni*
	*N*. *sandersi*	*N*. *venticola*, *P*. *laevis*
	Nemertina	*N*. *venticola*
	*Glycera* sp.	*Levinsenia* sp.
	*Lumbrineris* sp.	*Cirratulus* sp.
S_Gast	*B*. *guaymasensis*	Actinaria, *Hyalogyrina* sp.
	*B*. *hessleri*	*N*. *sandersi*, *Aphelochaeta* sp
S_Sib	*A*. *rosacea*	*L*. *guaymasensis*, Dorvilleids
V_Sib	*O*. *akessoni*	*P*. *grasslei*, *A*. *mammillatus*, *Cataegis* sp. juveniles
	*L*. *riftense*	*O*. *akessoni*, *P*. *bactericola*
V_VesA	*A*. *rosacea*	*Provanna* sp., *P*. *laevis*
	*N*. *sandersi*	*Provanna* sp., *P*. *laevis*, *A*. *rosacea*
	*Sigambra* sp.	*N*. *venticola*
	*B*. *guaymasensis*	*A*. *fauchaldi*, *A*. *rosacea*

Predation- Among the three reference, seven seep and six vent taxa identified as predators, only four appeared to rely on a specific prey (i.e. a nemertean, the polychaetes *Glycera* sp. and *Lumbrineris* sp. at seeps and *Sigambra* sp. at vents, Table 4). All other predators had multiple potential preys.

Co-occurring foundation species- Bivalve foundation species that co-occur at the three vesicomyid-dominant assemblages differed in their δ^13^C signatures with systematic enrichments of ~5‰ for solemyids (*A*. aff. *johnsoni*) compared with vesicomyids (*A*. *gigas*, *P*. *soyoae* and *C*. *pacifica*) (Table 5). Conversely, their δ^15^N signatures were not consistently different and value shifts varied (from 2 to 6‰). Siboglinid foundation species that co-occur at seep had comparable δ^13^C signatures, although their δ^15^N signatures differed with *E*. *spicata* being enriched by 5‰ compared with *L*. *barhami*. Finally, alvinellid foundation species that co-occur at vent showed comparable δ^13^C signatures, but their δ^15^N signatures differed, with *P*. *bactericola* being enriched by 3‰ compared with *P*. *grasslei*.

**Table 5:** Variability of carbon and nitrogen isotope ratios among co-occurring foundation species in the Guaymas vent and seep ecosystems.

Assemblage	δ^13^C, p_value	Foundation species	Shift	δ^15^ N, p_value	Foundation species	Shift
S_VesP	< 0.05	*A*. aff. *johnsoni* > *P*. *soyoae*, *C*. *pacifica*	5‰	< 0.05	*P*. *soyoae*, *C*. *pacifica* > *A*. aff. *johnsoni*	2 to 3‰
S_VesA	< 0.01	*A*. aff. *johnsoni* > *A*. *gigas*	6‰	ns	*A*. *gigas* = *A*. aff. *johnsoni*	-
V_VesA	< 0.01	*A*. aff. *johnsoni* > *A*. *gigas*	5‰	< 0.01	*A*. *gigas* > *A*. aff. *johnsoni*	6‰
S_Sib	ns	*L*. *barhami* = *E*. *spicata*	-	< 0.01	*E*. *spicata* > *L*. *barhami*	5‰
V_Alv	ns	*P*. *grasslei* = *P*. *bactericola*	-	< 0.01	*P*. *bactericola* > *P*. *grasslei*	3‰

### Inter-assemblage variability in faunal isotope ratios

#### δ^13^C signatures

Endosymbiotic species- Endosymbiotic taxa shared among assemblages had relatively similar δ^13^C signatures (Table 6). Both vesicomyid and solemyid endosymbiotic species had δ^13^C signatures related to thiotrophy using the CBB cycle and each species had constant δ^13^C values across assemblages. The single, undefined juvenile vesicomyid specimen found at the immediate periphery of V_Alv had a δ^13^C signature of -37‰, similar to other vesicomyids. Siboglinids had δ^13^C signatures close to the ones of thiotrophy using the rTCA cycle and there were significant differences in δ^13^C signatures between seep and vent assemblages, with an enrichment of 5‰ at vents.

**Table 6:** Variability of carbon and nitrogen isotope ratios of endosymbiotic species among assemblages in the Guaymas vent and seep ecosystems.

δ^13^C	Taxon	p_value	Assemblages	Shift
	*A*. *gigas*	ns	S_VesA = V_VesA	-
	Vesicomyids	ns	S_VesP = S_VesA = V_VesA	-
	*A*. aff. *johnsoni*	ns	S_VesP = S_VesA = V_VesA	-
	Siboglinids	0.01	V_Sib > S_Sib	5‰
δ^15^N	Taxon	p_value	Assemblages	Shift
	*A*. *gigas*	001	S_VesA >V_VesA	5‰
	Vesicomyids	< 0.001	S_VesA > S_VesP > V_VesA	5 to 9‰
	*A*. aff. *johnsoni*	0.02	S_VesA > S_VesP > V_VesA	9 to 16‰
	Siboglinids	0.03	S_Sib *(E*. *spicata*) > S_Sib (*L*. *barhami*), V_Sib (*R*. *pachyptila*)	6‰

Heterotrophic species- Heterotrophic taxa that were common to several assemblages within each ecosystem showed similar δ^13^C ranges (Table 7). For species common to seeps and vents, a consistent ^13^C enrichment was found at vents, with δ^13^C differences reaching values as high as 20‰.

**Table 7:** Variability of carbon isotope ratios of heterotrophic species among assemblages in the Guaymas vent and seep ecosystems.

Ecosystem	Taxon	p_value	Assemblages	δ^13^C shift
Seep	*E*. *lomana*	ns	S_VesP = S_VesA	-
Seep	*Parougia* sp.	ns	S_VesA = S_Gast	-
Seep	*Actinaria* undet	ns	S_VesA = S_Gast	-
Seep	*Ophiura* sp.	0.005	S_VesA > S_VesP, S_Gast	2‰
Seep	*Cirratulus* sp.	0.001	S_VesA > S_VesP	4‰
Vent	*P*. *grasslei*	ns	V_Alv = V_Sib	-
Seep,Vent	*N*. *grasslei*	0.003	V_VesA > S_VesP, S_Mat, S_VesA, S_Gast	9‰
Seep,Vent	*R*. *diploura*	0.05	V_VesA > S_VesA	4‰
Seep,Vent	*P*. *laevis*	0.001	V_VesA > S_VesP, S_VesA	4 to 10‰
Seep,Vent	*A*. aff. *fauchaldi*	< 0.001	V_Alv, V_Sib, V_VesA > S_Gast, S_VesA	16 to 20‰
Seep,Vent	*N*. *sandersi*	0.1	V_VesA > S_VesA	13‰
Seep,Vent	*B*. *Sandersi*	0.1	V_Alv >S_Sib	1‰
Seep,Vent	*B*. *guaymasensis*	0.07	V_VesA > S_VesA, S_Gast, S_Mat	7‰
Seep,Vent	*Terrebellid* undet	< 0.001	V_VesA > S_Gast, S_Sib, S_VesP	5 to 11‰
Seep,Vent	*M*. *alvisca*	0.02	V_VesA > S_Sib	16‰

#### δ^15^N signatures

**Fig 6:**
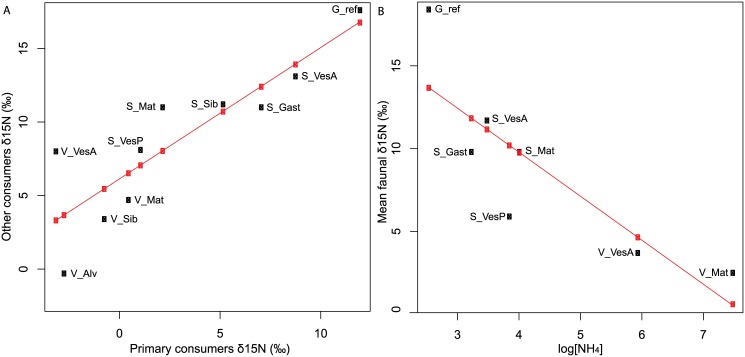
Spatial variability of nitrogen isotope ratios. (A) Regression between mean δ^15^N signatures of primary consumers and the remaining consumers for all assemblages (p_value = 0.001, adjusted R^2^ = 0.73), (B) Regression between mean faunal δ^15^N signatures and log-transformed ammonium concentrations for soft-sediment assemblages (p_value = 0.02, adjusted R^2^ = 0.66).

Endosymbiotic taxa common to several assemblages had δ^15^N values that differed greatly within and between ecosystems (Table 6). We therefore tested whether the δ^15^N signatures of primary consumers relying specifically on chemoautotroph microbes (endosymbiotic species and bacterivores) reflected the δ^15^N baseline shifts among assemblages. The correlation among the mean δ^15^N signatures of these primary consumers and the remaining members of the communities was highly significant (p< 0.001, adjusted R^2^ = 0.73) (Fig 6). Thus, the high δ^15^N signature variations observed among assemblages at the base of the food web were also reflected in the associated faunal communities. In addition, log-transformed NH_4_^+^ concentrations available from soft sediments were negatively correlated with the mean faunal δ^15^N signatures (p< 0.02, adjusted R^2^ = 0.66, Fig 6).

However, no baseline correction was applied to faunal δ^15^N signatures because there was strong variability among co-occurring foundation species related to endosymbiotic or bacterivore primary consumers reflecting multiple δ^15^N baselines (see 3.1.3.). Furthermore, consumers may rely not only on chemosynthetic production, but also on a non-negligible proportion of photosynthetic OM, especially for taxa typical of the background area, which had highly enriched δ^15^N values. Thus, interpretations of δ^15^N signature variability of taxa common to several assemblages were limited and likely involve baseline rather than trophic level variation.

### Food-web structure

Food-web metrics at the community level require that the overall δ^13^C signature ranges of potential sources among assemblages be comparable. The reference assemblage, which has a single basal source (photosynthesis-derived POM) was thus only included in the representation of SEAc values. All seep and vent assemblages have similar potential sources with comparable overall δ^13^C signature ranges, at least within ecosystems. The only exception was *Parougia* sp. found in two assemblages (S_Gast and S_VesA). These dorvilleids had extremely depleted isotopic signatures characteristic of specialists feeding on methanotrophic archaea involved in AOM consortia (~ -80‰) and were thus excluded from our food-web structure analysis. Between ecosystems, comparisons may be biased by variation in δ^13^C signatures with regard to methane. The overall δ^13^C signature range of basal sources was estimated from 50 to 35‰ at seeps and from 40 to 25‰ at vents. Therefore, community metric comparisons were carried out within and between chemosynthetic ecosystems, in full awareness of the potential bias associated with seep and vent comparisons.

We assessed food web complexity at two levels. First, to describe their overall extent, the comparisons of SEAc, SEAb, CR and NR among assemblages were based on all community members. Then, to describe the degree of trophic specialisation and redundancy among communities, the comparisons of CD and CVNND among assemblages focused on deposit-feeder communities (including detritivores and bacterivores) that were represented in all assemblages. Mean values for each metrics were statistically compared using posterior distributions of the estimates.

#### Bayesian metrics

**Fig 7:**
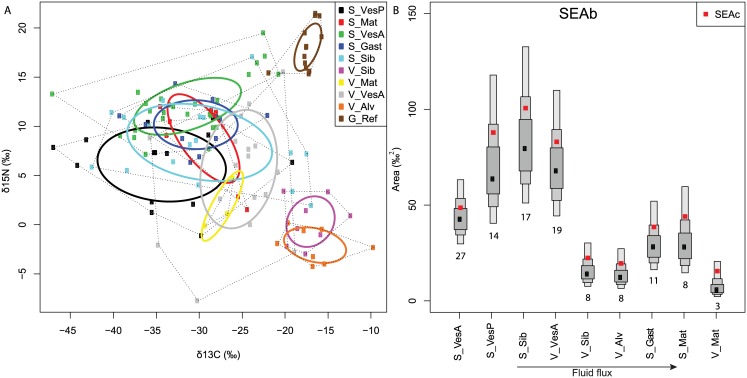
Standard ellipse areas. (A) Solid lines enclose the standard ellipse area (SEAc), containing ca. 40% of the data. (B) Density plots showing the credibility intervals of the Bayesian standard ellipse areas (SEAb). Black squares are the mode SEA, and boxes indicate the 50, 75 and 95% credible intervals. Red squares are the sample-size-corrected SEA (SEAc). Numbers below boxes give the number of invertebrate species sampled.

SEAs- Standard ellipse areas (SEAc and SEAb) refer to the overall extents of food webs. SEAc distributions showed differences among assemblages due to the variable nature of the nitrogen and carbon basal sources (Fig 7). SEAb globally differentiated the food webs of low fluid-flux assemblages with high trophic diversity (seeps: siboglinids and vesicomyids; vents: vesicomyids) from high fluid-flux assemblages (seeps: gastropods and mats; vents: siboglinids, alvinellids and microbial mats). Among assemblages common to seeps and vents, S_Mat had higher SEAb than V_Mat (p = 0.02), whereas S_VesP and V_VesA had comparable SEAb, both higher than S_VesA (p< 0.05).

**Fig 8:**
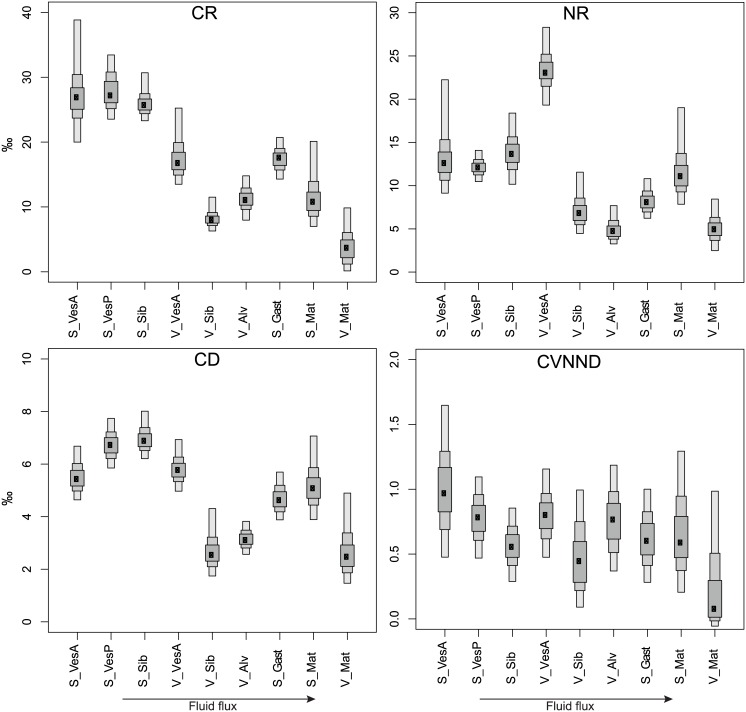
Bayesian results for the δ^13^C range (CR), δ^15^N range (NR), mean distance to centroid (CD) and the coefficient of variation of the nearest neighbour distance (CVNND). Black dots are the modes and boxes indicate the 50, 75 and 95% credibility intervals, from wider to thinner.

CR, NR- Carbon range (CR) reflects the niche diversification at the base of food webs. CR followed the same global pattern as SEAb with higher CR at low than high fluid-flux assemblages (Fig 8). Among common assemblages, microbial mats and vesicomyids had higher CR at seeps than vents (p = 0.03 and < 0.05, respectively).

Nitrogen range (NR) typically indicates the number of trophic levels. In our study, NR likely reflects not only trophic levels, but also the presence of multiple δ^15^N baselines within assemblages (see 3.2.2). Unlike CR, the NR pattern differed from the SEAb one (Fig 8). The seep mat assemblage had higher NR values than the vent one (p = 0.02), whereas seep vesicomyids had lower NR values than vent assemblage (p< 0.05).

CD- Distances to the centroid (CD), indicative of trophic specialisation, differentiated food webs of low fluid-flux assemblages (characterised by high trophic specialisation) from high fluid-flux assemblages (Fig 8). Seep mat had higher CD than vent one (p = 0.04) whereas S_VesA and V_VesA had comparable CDs that were both lower than S_VesP (p = 0.06 and 0.09, respectively).

CVNND- Although the coefficients of variation of the nearest neighbour distance (CVNND), which indicate trophic redundancy within communities, poorly differentiated the assemblages, there was a general trend showing an increase in trophic redundancy at high fluid-flux assemblages (i.e., lower CVNND values, Fig 8). Seep mat had higher CVNND values than vent mat (p = 0.01) whereas values are comparable among seep and vent vesicomyid assemblages.

#### Relationships between food-web metrics and environmental conditions

**Fig 9:**
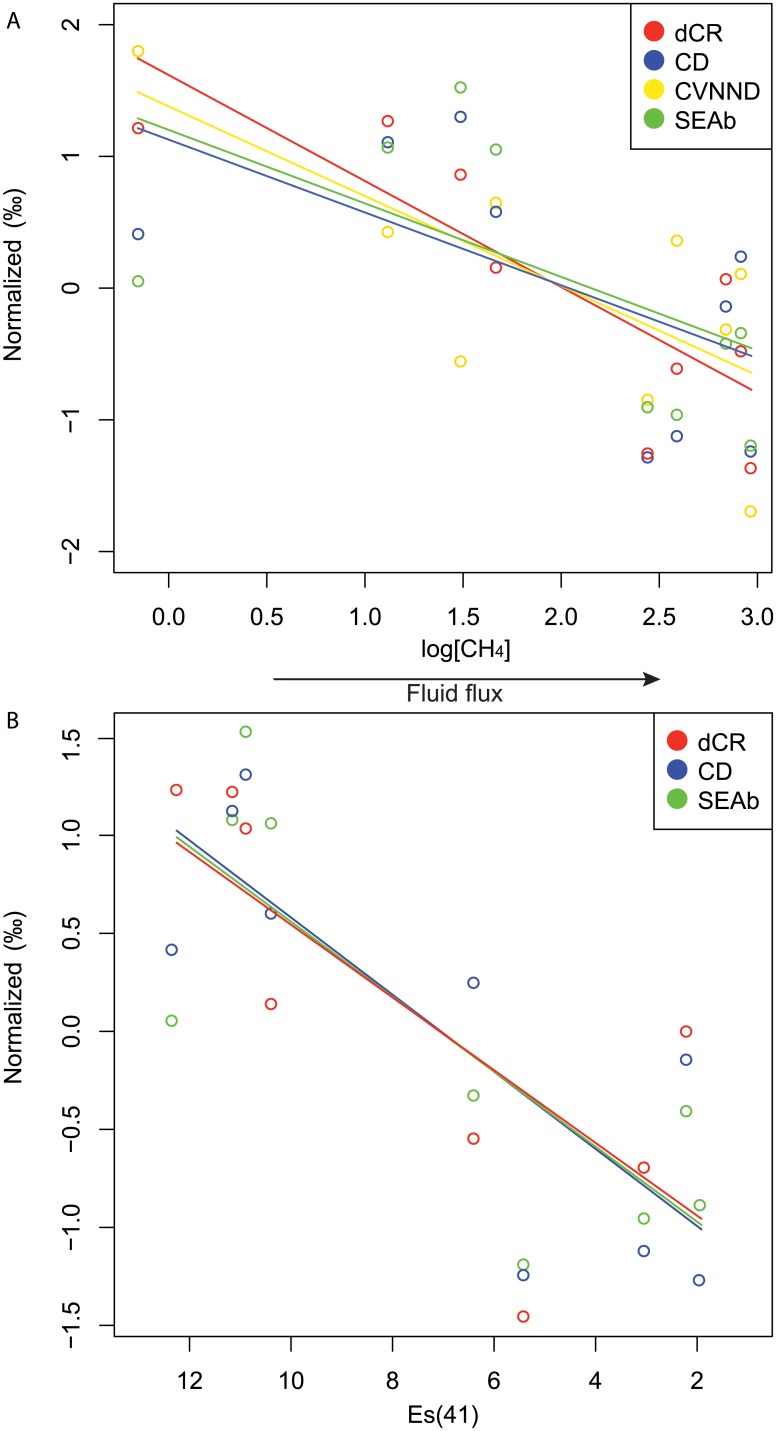
Spatial variability of food web structure. (A) Regression lines with respect to methane concentrations for dCR (p = 0.004, adjusted R^2^ = 0.67), SEAb (p = 0.09, adjusted R^2^ = 0.25), CD (p = 0.08, adjusted R^2^ = 0.28) and CVNND (p = 0.02, adjusted R^2^ = 0.47) and (B) Regression lines with respect to alpha diversity (Es(41)) for dCR (p = 0.01, adjusted R^2^ = 0.58), SEAb (p = 0.01, adjusted R^2^ = 0.59), CD (p = 0.007, adjusted R^2^ = 0.62).

Different lines of evidence suggest a relationship between food-web metrics and methane concentration (common proxy for seep and vent fluid fluxes) whereas no relation to temperature (vent-specific proxy) was found. At the 0.10 significance level, negative correlations with methane concentrations were found for SEAb (p = 0.09, adjusted R^2^ = 0.25), CR (p = 0.004, adjusted R^2^ = 0.67), CD (p = 0.08, adjusted R^2^ = 0.28) and CVNND (p = 0.02, adjusted R^2^ = 0.47), but no correlations were found for NR. In addition, several food-web complexity metrics were significantly correlated with macrofaunal alpha diversity (ES(41)), including SEAb (p = 0.01, adjusted R^2^ = 0.59), CR (p = 0.01, adjusted R^2^ = 0.58) and CD (p = 0.007, adjusted R^2^ = 0.62), but not for NR or CVNND. Regression lines showed a linear decrease in food-web complexity along increasing logarithmic methane concentrations and decreasing ES(41), with no distinction between the two ecosystems (Fig 9).

## Discussion

This study provides the first local-scale comparison of food-web structures across seep and vent ecosystems, enhancing our knowledge on biosynthetic pathways sustaining food webs, species trophic ecology (i.e. trophic guilds and biotic interactions) and on patterns of food-web complexity along fluid-flux gradients.

### Basal source contributions

The Guaymas Basin benefits from the high biological productivity in surface waters, producing a particulate organic carbon flux of ~2400 mg C.m^-2^.d^-1^ and a sedimentation rate of 2.7 mm.y^-1^ [89–91]. However, consistent with the main features of deep-sea chemosynthetic ecosystems, photosynthesis-derived organic matter was identified as a minor energy source in Guaymas seep and vent food webs [77, 92, 93]. The few taxa that predominantly rely on this basal source were mostly deposit-feeder polychaetes that have been shown to selectively feed on phytodetritus [44]. Interestingly, these taxa were only found in the five vent or seep assemblages featuring the lowest fluid fluxes and belong to families that are related to typical deep-sea background fauna [44, 94]. Therefore, although background taxa may colonise seep and vent low fluid-flux habitats, they do not appear to feed on the local chemosynthetic production. Conversely, all taxa endemic to seeps and vents within this study were predominantly sustained by endogenous microbial production despite the organic-rich sedimentary context of the Guaymas Basin.

While potential endogenous microbial sources sustaining Guaymas seep and vent food webs are similar, their contributions were shown to vary within and especially between ecosystems:

1. Thiotrophs using the Calvin-Bensin-Bassham (CBB) cycle dominated in seeps and vents, whereas thiotrophs using the reductive tricarboxylic acid (rTCA) cycle dominated only in vents. Accordingly, CBB thiotrophs are commonly described in seep and vent ecosystems, whereas rTCA thiotrophs are considered as being typical of vent ecosystems [95]. Nonetheless, seep siboglinid tube worms and their commensal/parasitic polychaetes predominantly relied on rTCA-cycle thiotrophs thus providing additional support for recent findings that argue for a non-negligible role of rTCA thiotrophy in seeps [54].
2. The contribution of methanotrophy to both seep and vent food webs was non-negligible but appeared higher at seeps than at vents. Additionally, one species at seeps (*Parougia* sp. Dorvilleids) was related to a specialised archivorous diet, reflecting the consumption of archaeal-bacterial aggregates involved in the anaerobic oxydation of methane (AOM) [66]. While the same dorvilleid species was found in Guaymas vents [26], isotope signatures were not measured for lack of a sufficient number of specimens, archivory in our vent assemblages thus remains to be determined.
3. Petroleum-derived organic matter contributed to vent food webs and was even one of the dominant food sources, especially in the vesicomyid assemblage where all organisms with the exception of vesicomyids may rely on this basal source. Further evidence includes the direct observation of seeping petroleum during sediment sampling in vesicomyid habitat (author’s obs.). Accordingly, previous studies have shown that petroleum-derived carbon can be directly incorporated into fresh microbial biomass and sustain heterotrophic organisms at Guaymas vents [96, 97]. High contributions of petroleum-derived organic matter to food webs have also been reported in highly polluted areas or oil seeps [98–101]. At Guaymas seeps, although microbial genes involved in hydrocarbon catabolism have been reported [102], petroleum contribution was not detected within the food webs, possibly reflecting its lower availability than at vents.

The relative contributions of basal sources to Guaymas vent and seep food webs differed despite similar hydrogen sulphide and methane concentration ranges [26]. Seep and vent geochemical discrepancies (e.g. temperature, metals) may account for differences in community structure of microbial primary producers. For example, microbial communities involved in AOM or anammox have been shown to differ between seeps and vents [26, 103]. Therefore, the presence of common seep and vent species [26], raises questions regarding their reliance on specific basal sources. Although common endosymbiotic species had comparable δ^13^C signatures across ecosystems, heterotrophic species showed strong differences (shifts up to 20‰), consistent with high contributions of ^13^C-enriched carbon sources at vents (petroleum-derived production and rTCA-based thiotrophy), and ^13^C-depleted sources at seeps (methanotrophy and CBB thiotrophy). The weak trophic links of heterotrophic taxa to the metabolic diversity of chemosynthetic primary producers may be a key to their adaptation to environmental variability across ecosystems.

Beyond carbon sources, contributions of inorganic nitrogen sources to primary production within chemosynthetic food webs remain poorly defined [104]. The δ^15^N signatures of species were particularly variable among assemblages and reflected δ^15^N baseline variations. Interestingly, mean faunal δ^15^N signatures within assemblages were negatively correlated with log-transformed ammonium concentrations. Ammonium, which is produced during microbial organic matter degradation, is usually ^15^N-depleted [81, 105]. Furthermore, in the Guaymas Basin and especially at vents due to the thermocatalytic percolation of organic matter, ammonium concentrations reach exceptionally high values that may contribute to the depleted δ^15^N signatures. During ammonium uptake, high concentrations may lead to the full expression of the theoretical fractionation of ~30‰ relative to the substratum [80, 81]. In addition to the inter-assemblage variability, co-occurring endosymbiotic or bacterivorous primary consumers showed strong δ^15^N differences, reflecting multiple baselines, which have been related to ammonium concentrations gradients within sediments. Overall, our results indicate that primary producers depend greatly on local nitrogen sources rather than on water column nitrates [45] and that their δ^15^N signatures can vary greatly with local biogeochemical processes between and within habitats [79].

### Species trophic ecology

The characterisation of Guaymas seep and vent food webs considered a total of 46 taxa at seeps, including 6 endosymbiotic species and 29 at vents, including 3 endosymbiotic species. In accordance with other studies (reviewed in [7]), endosymbiotic species that dominated the biomass [26] did not significantly contribute to the diet of other taxa, except for a few seep parasitic or commensal species. These relationships were suggested for a new species of copepod and nautiliniellid polychaete on solemyid and vesicomyid bivalves as well as phyllodocid and polynoids polychaetes on siboglinid tubeworms. Many copepods have already been described as parasites in chemosynthetic ecosystems [106]. The relationship between nautiliniellid polychaetes and their bivalve hosts remains unclear [107] as they have been suggested to consume bivalve tissue, mucus, gametes pseudofaeces [78] or suspended particles resulting from gill ciliary activity [108]. A parasitic relationship of phyllodocids on sibolginids has already been suggested based on stable isotope ratios that was further supported by the observation of phyllodocids living in a matrix of tubes affixed to the top of the tubeworms, along with the presence of a worm-blood-resembling substance in their guts [78]. Finally, although some polynoid species have been identified as siboglinid predators at vents [109, 110], in our study, the nature of the relationship between *B*. *sandersi* and siboglinids remains undefined. *B*. *sandersi* isotope signatures indicated that it may rely only partially on siboglinid tissue, along with other food sources such as chemoautotrophic microbes. Therefore, more studies are needed to identify diets of potential commensal/parasitic species and to characterise the nature of their relationships (facultative or obligate) with their hosts. In addition to commensal/parasitic species, several predators have been identified within seep and vent communities. Among them, those apparently feeding on specific prey were rare. Most predators were relying on multiple prey species thus being generalists rather than specialists, as already proposed for Juan de Fuca vents [36] and for Gulf of Mexico cold seeps [111]. While potential parasitic/commensal and predator taxa were poorly represented, the heterotrophic fauna was mainly composed of detritivorous and bacterivorous taxa. These results together with the low number of specialist predators support the hypothesis that food webs in chemosynthetic ecosystems are not organised along specific predator-prey links, but rather through weak trophic relationships among co-occurring species [111, 112].

In addition to direct trophic relationships among species, the analysis of stable isotope ratios may further reveal trophic partitioning among co-occurring and potentially competitive species. Indeed, species coexistence in chemosynthetic ecosystems and especially between foundation species is most likely driven by species sorting [113]. Species sorting is related to the classical niche theory [114] whereby species co-existence draws on differentiation in resource use, spatial partitioning of the same resource or density-dependent predation risks [115]. Within seep and vent ecosystems in the Guaymas Basin, several taxonomically and functionally closely related foundation species co-occur (i.e. siboglinids at seeps, alvinellids at vents, vesicomyids and solemyids at both seeps and vents). Comparison of their stable isotope ratios can help to test niche differentiation driven either by trophic or spatial partitioning. As discussed above, predation appears to be of minor importance in chemosynthetic ecosystems and thus unlikely to foster the co-existence of competing species.

At seeps, two siboglinid species, *E*. *spicata* and *L*. *barhami*, co-occured. Although their δ^13^C signatures were comparable, their δ^15^N values differed strongly, with *E*. *spicata* being more enriched (by ~5 ‰) than *L*. *barhami*. A previous study also found similar δ^15^N shifts between two seep siboglinid taxa (*E*. *laminata* and *Lamellibrachia* sp. 1) with an enrichment of ~3‰ [116]. The δ^15^N signature differences between the two species may either reflect inorganic nitrogen source partitioning (e.g. nitrate, ammonium), spatial partitioning of nitrogen uptake (interstitial pore waters through their roots or seawater through their gills), or distinct fractionation factors by their endosymbionts [116, 117]. *E*. *spicata* and *L*. *barhami* symbionts in the Guaymas Basin belong to the same phylotype, which can be characterised by several subtypes [101, 118]. The relative proportions of these subtypes can vary according to environmental conditions, but they are similar in the two species when they co-occur. Therefore, no host-specific relationship has been identified for endosymbionts among siboglinids. The δ^15^N signature discrepancy observed in our study is thus more likely linked to nitrogen source partitioning and/or to the spatial segregation of the compartments from which siboglinids extract nitrogen sources.

At vents, the two alvinellid species *P*. *grasslei* and *P*. *bactericola* co-occured. Both species are bacterivorous specialists [44, 119] and had comparable δ^13^C but different δ^15^N signatures. The dominant species *P*. *grasslei* [26] showed a depleted δ^15^N signature (by ~5‰) in comparison with *P*. *bactericola*. A previous study of spatial isotope variability among three sympatric alvinellid species, *P*. *palmiformis*, *P*. *sulfincola* and *P*. *pandorae* on the Juan de Fuca ridge showed that highest or lowest δ^15^N signatures were not consistently associated with the same species across assemblages [16]. Thus, these differences were not likely due to interspecific differences in isotope fractionations during food assimilation, but rather to food-source partitioning and/or spatial segregation. The same process may be at work in the Guaymas Basin. Furthermore, *P*. *bactericola* occasionally feeds on larger suspended aggregates through its two large tentacles whereas *P*. *grasslei* may be limited to deposit feeding owing to its smaller tentacles [119]. *P*. *bactericola* is also usually embedded in bacterial mat-covered sediments while most other alvinellid species are attached to a hard substratum [44]. In this study, the two species were found in a hard substratum habitat covered by thick mucus/microbial mats and may therefore also be affected by small scale spatial segregation.

Finally, at seep and vent ecosystems, vesicomyids and solemyids, endosymbiotic and potentially competing bivalves, co-occurred. The *A*. aff. *johnsoni* solemyid δ^13^C signature was consistently depleted (by ~5‰) in comparison with vesicomyids. Therefore, these interspecific δ^13^C variations likely reflect the presence of specific types of symbionts characterised by different fractionation factors during the assimilation of CO_2_. Furthermore, the vesicomyid and solemyid symbionts belong to very distant phylogenetic groups [58]. On the other hand, the differences in δ^15^N signatures between vesicomyids and solemyids were also high but inconsistent between assemblages (δ^15^N values were either higher or equal in vesicomyids), suggesting the use of different nitrogen sources by their symbionts and/or spatial segregation within the assemblages. Solemyids are usually found in deeper sediment layers than vesicomyids [120], where high concentrations of depleted nitrogen source (e.g. ammonium) result from organic matter mineralisation. In addition, the isotope fractionation factors of their endosymbionts may also vary according to the concentrations of inorganic nitrogen sources with high concentrations potentially inducing strongly depleted δ^15^N signatures [80]. Interestingly, strongest difference of δ^15^N signatures between solemyids and vesicomyids (~6‰) was linked to the vent assemblage in which ammonium concentrations reached a maximum along the 10-cm depth layers (i.e., from 30 μM at 0–2 cm to 380 μM at 10 cm) [26]. The second greatest difference (~3‰) was found in seep assemblages where ammonium concentrations reached values of 50 μM (from 10 μM at 0–2 cm to 50 μM at 10 cm). Finally, the assemblage in which vesicomyid and solemyid δ^15^N signatures were not significantly different showed no increase in ammonium concentrations (~30 μM along the whole core). ^15^N depletion in solemyids is thus consistent with the fact that they dig deeper in the sediments than vesicomyids and strongly supports the hypothesis of spatial segregation in resource uptake between the two species. All together, these results provide evidence that these sympatric species either benefit from food source partitioning or from spatial habitat segregation that may decrease their competitive interactions, in agreement with the species sorting model [113]. Overall, while predation does not seem to play a significant role in structuring vent and seep communities, with predators being rather rare and generalists, competition might foster niche diversification and play a significant role in the structure and functioning of vent and seep ecosystems.

### Food-web structural complexity

At vent and seep ecosystems, previous studies have suggested that food-web complexity vary with fluid chemistry [95, 111, 121–123]. In our study, variations in the relative dominance of trophic guilds did not show any clear pattern related to fluid-flux settings or ecosystems. Species trophic strategies did not appeared as a criterion of selection along fluid gradients. However, significant relationships were found between Bayesian food-web metrics and methane concentrations, a consistent proxy for fluid flow across assemblages and ecosystems. Those indices, based on community δ^13^C - δ^15^N spaces allowed to integrate species ecological niche taking into account both trophic and habitat components thus illustrating the realised trophic niche [84, 85]. Congruent patterns across ecosystems suggested that similar processes drive the functioning of vents and seeps. Low fluid-flux assemblages (seeps: siboglinids and vesicomyids, vents: vesicomyids) had more complex food webs, due to higher trophic diversity and specialisation, but lower trophic redundancy than high fluid-flux assemblages (seeps: gastropods and microbial mats, vents: siboglinids, alvinellids and microbial mats). All these metrics co-varied with niche diversification at the base of food webs (δ^13^C signature range). The variability in food-web complexity among assemblages thus seems closely related to the interplay of food source diversity and partitioning among species. In chemosynthetic ecosystems, microbial primary production is usually positively correlated with fluid flux, which provides the reduced compounds sustaining microbial production [124–126]. Nevertheless, microbial diversity usually decreases with increased fluid flux [124, 127]. Therefore, fauna at low fluid-flux assemblages may benefit from less abundant, but more diverse food sources compared to high fluid-flux assemblages. Concurrently, lower productivity may also involve more intense interspecific competition among taxa, which in turn results in higher trophic specialisation and lower redundancy. Furthermore, alpha diversity was also positively correlated to food web complexity suggesting that the establishment of diverse communities may require strong niche partitioning. In low fluid-flux assemblages where alpha diversity was maximal, trophic and/or spatial partitioning was for example evidenced between endemic species and background migrant (section 4.1) as well as between sympatric species (section 4.2).

Beyond the patterns of food-web complexity along fluid-flux gradients, additional variability among food webs can be attributed to interacting factors such as the engineering role of foundation species. The estimation of their respective roles is often a challenge in chemosynthetic ecosystems because the distribution of foundation species is strongly correlated with fluid flux [120, 128, 129]. In our study, the presence of two distinct vesicomyid assemblages dominated by either *A*. *gigas* or *P*. *soyoae* and characterised by comparable fluid-flux settings (methane concentrations) in the seep ecosystem offered an opportunity to assess their respective roles in structuring the food web. Although *P*. *soyoae* may have stronger authigenic effects (i.e. habitat provision), *A*. *gigas* may exhibit stronger allogenic effects (i.e. bioturbation) [26, 130]. Enhanced bioturbation by *A*. *gigas* may potentially increase sulphate penetrations and thus indirectly promote sulphide production via the anaerobic oxidation of methane (AOM) in deeper sediment layers, as already suggested for other vesicomyid clams [131, 132]. Although food webs showed comparable trophic redundancy and δ^13^C and δ^15^N signature ranges, lower trophic diversity and specialisation were associated with *A*. *gigas* assemblage compared with that of *P*. *soyoae*. Interestingly, the *A*. *gigas* assemblage had higher macrofaunal alpha diversity and density compared with *the P*. *soyoae* communities [26]. Therefore, higher microbial productivity in *A*.*gigas* assemblages may partly release species from trophic competition, thus allowing colonisation by a dense and diverse community without high trophic diversity and specialisation. These results highlight the engineering roles that foundation species may play on the functional heterogeneity between assemblages. Furthermore, it reinforces the structuring role of competitive interactions for species coexistence (i.e. species sorting) in low fluid-flux assemblages.

**Fig 10:**
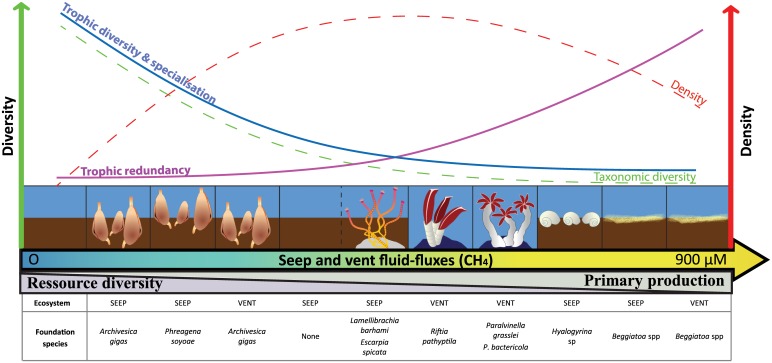
Updated conceptual diagram of faunal community structure and food-web patterns along fluid-flux gradients within Guaymas seep and vent ecosystems. Adapted from [25] and modified from [26].

Overall, food-web complexity may be structured through the interplay between the availability and diversity of food sources, biotic interactions (competition, facilitation), and abiotic pressures. We hypothesise that the weak environmental constraints and high basal source diversity of low fluid-flux settings foster colonisation by numerous taxa and that their coexistence, in a context of lower primary productivity, may require high niche partitioning, resulting in high trophic diversity and specialisation, but low redundancy (Fig 10). In contrast, the strong environmental constraints and low basal source diversity in high fluid-flux settings may limit consumer colonisation to a few tolerant taxa that are functionally similar and/or potentially released from competitive pressure because primary production is assumed to be high, resulting in low food-web complexity and high trophic redundancy. Therefore, seep and vent functioning contrast with non-chemosynthetic ecosystems in which food web complexity is usually higher at highly productive areas [133]. Here, the environmental constraints along fluid-flux gradients appeared to overwhelm the beneficial effect of primary production on food webs. Nonetheless, our study is consistent with the global marine habitat patterns where food-web complexity, together with the structuring role of competitive interactions, decreases along gradients of increasing environmental constraints [134–139]. Finally, although environmental conditions (physico-chemistry, dominant basal sources) varied between seep and vent ecosystems, their food web structure was highly similar with a non-significant role of vent-specific factors. In addition to the high trophic flexibility of seep and vent common species, ecosystem functioning similarity suggested that species might occupy equivalent ecological niches across ecosystems. These results suggest that ecological niches are not specifically linked to the nature of fluids and thus provide further support to the hypothesis of continuity among deep-sea chemosynthetic ecosystems [26, 27].

## Conclusion

This study of Guaymas seep and vent food webs offers one of the first opportunities to assess and compare the functioning of these two deep-sea chemosynthetic ecosystems. Both seep and vent food webs did not appeared to be structured along predator-prey links but rather through weak tropic links among co-occurring species.

Our results suggest that in seeps and vents, food-webs may similarly be shaped through the interplay between the availability and diversity of food sources, biotic interactions (competition, facilitation), and abiotic pressures. The food web structure assessed through Bayesian metrics showed a decrease of food-web complexity that was consistent along a gradient of increasing fluid-flux intensity crossing seep and vent assemblages. This pattern is hypothesized to result from two set of processes:

1. In low fluid-flux settings, weak environmental constraints and high basal source diversity may foster colonisation by numerous taxa which coexistence, in a context of lower primary productivity, may require high niche partitioning, resulting in high trophic diversity and specialisation, but low redundancy. Examples of niche differentiation either through food source partitioning or spatial segregation were evidenced, particularly in low fluid-flux settings, which reflected the importance of species-sorting processes in chemosynthetic ecosystems.
2. In high fluid-flux settings, strong environmental constraints may overwhelmed the benefit of high primary productivity by limiting basal source diversity, consumer colonisation to a few tolerant taxa that are functionally similar, and the structuring role of biotic interactions, leading to an overall reduction of food web complexity and a higher trophic redundancy.

Finally, while environmental conditions discriminated seeps and vents (e.g. physico-chemistry, dominant basal sources), food-web complexity pattern did not differentiate these two ecosystems. These results provide support for a clear continuity between cold seeps and hydrothermal vents in the Guaymas Basin with faunal communities showing both structural and functional patterns that are consistent across ecosystems.

## Supporting Information

S1 Table Mean stable isotope composition (expressed in ‰) of the species, sediment and methane samples from the ten studied assemblages. Standard deviations are given in parentheses. Consumers trophic guilds are classified, according to the literature and our results, as S: Symbiont bearing, B: Bacterivore and Archivore, D: Detritivore/Scavenger, P: Predator, C: Commensalist/Parasitic, followed by d: deposit feeder or grazer, s: suspension feeder. Bacterivore and Archivore are considered as specialist detritivores. Values in grey correspond to peripheral samples that are not included within our study.
(DOCX)

S1 Fig Illustration of the copepod species nov deeply attached to *Acharax* aff. *johnsoni* gill lamellae (A) and zoom on the copepod head (B). (DOCX)
