# Supplementary material for: Food-Web Complexity in Guaymas Basin Hydrothermal Vents and Cold Seeps
Source: PLoS One. 2016 Sep 28;11(9):e0162263. doi: 10.1371/journal.pone.0162263 (PMC5040445; doi:10.1371/journal.pone.0162263)
Supplement: S1 Table — Standard deviations are given in parentheses. Consumers trophic guilds are classified, according to the literature and our results, as S: Symbiont bearing, B: Bacterivore and Archivore, D: Detritivore/Scavenger, P: Predator, C: Commensalist/Parasitic, followed by d: deposit feeder or grazer, s: suspension feeder. Bacterivore and Archivore are considered as specialist detritivores. Values in grey correspond to peripheral samples that are not included within our study. (DOCX) [file pone.0162263.s001.docx]

**S1 Table. Mean stable isotope composition (expressed in ‰) of the species, sediment and methane samples from the ten studied assemblages.** Standard deviations are given in parentheses. Consumers trophic guilds are classified, according to the literature and our results, as S: Symbiont bearing, B: Bacterivore and Archivore, D: Detritivore/Scavenger, P: Predator, C: Commensalist/Parasitic, followed by d: deposit feeder or grazer, s: suspension feeder. Bacterivore and Archivore are considered as specialist detritivores. Values in grey correspond to peripheral samples that are not included within our study.

|  |  |  | **G_ref** | | | **S_VesP** | | | **S_VesA** | | | **S_Mat** | | | **S_Gast** | | | **S_Sib** | | | **V_VesA** | | | **V_Mat** | | | **V_Alv** | | | **V_Sib** | | |
| --- | --- | --- | --- | --- | --- | --- | --- | --- | --- | --- | --- | --- | --- | --- | --- | --- | --- | --- | --- | --- | --- | --- | --- | --- | --- | --- | --- | --- | --- | --- | --- | --- |
| **Taxa** | **Trophic strategy** | **Reference** | δ^13^C | δ^15^N | n | δ^13^C | δ^15^N | n | δ^13^C | δ^15^N | n | δ^13^C | δ^15^N | n | δ^13^C | δ^15^N | n | δ^13^C | δ^15^N | n | δ^13^C | δ^15^N | n | δ^13^C | δ^15^N | n | δ^13^C | δ^15^N | n | δ^13^C | δ^15^N | n |
| **Foundation species** |  |  |  |  |  |  |  |  |  |  |  |  |  |  |  |  |  |  |  |  |  |  |  |  |  |  |  |  |  |  |  |  |
| **Vesicomyidae** |  |  |  |  |  |  |  |  |  |  |  |  |  |  |  |  |  |  |  |  |  |  |  |  |  |  |  |  |  |  |  |  |
| *Archivesica gigas* | S | [[1](#_ENREF_1)] |  |  |  |  |  |  | -36.1 (0.2) | 7.1 (0.5) | 3 |  |  |  |  |  |  |  |  |  | -34.7 (2.5) | -2.1 (1.2) | 11 |  |  |  |  |  |  |  |  |  |
| *Phreagena soyoae* | S | [[2](#_ENREF_2)] |  |  |  | -35.4 (0.9) | 1.2 (1.5) | 13 |  |  |  |  |  |  |  |  |  |  |  |  |  |  |  |  |  |  |  |  |  |  |  |  |
| *Calyptogena pacifica* | S | [[1](#_ENREF_1)] |  |  |  | -35.4 (0.6) | 2.3 (0.9) | 5 |  |  |  |  |  |  |  |  |  |  |  |  |  |  |  |  |  |  |  |  |  |  |  |  |
| Vesicomyid juvenile | S | This study |  |  |  |  |  |  |  |  |  |  |  |  |  |  |  |  |  |  |  |  |  |  |  |  | -35.6 | -4.4 | 1 |  |  |  |
| **Siboglinidae** |  |  |  |  |  |  |  |  |  |  |  |  |  |  |  |  |  |  |  |  |  |  |  |  |  |  |  |  |  |  |  |  |
| *Riftia pachyptila* | S | [[3](#_ENREF_3)] |  |  |  |  |  |  |  |  |  |  |  |  |  |  |  |  |  |  |  |  |  |  |  |  |  |  |  | -12.3 (0.4) | 0.9 (1.0) | 6 |
| *Lamellibrachia barhami* | S | [[4](#_ENREF_4)] |  |  |  |  |  |  |  |  |  |  |  |  |  |  |  | -16.9 (2.5) | 1.9 (1.2) | 3 |  |  |  |  |  |  |  |  |  |  |  |  |
| *Escarpia spicata* | S | [[4](#_ENREF_4)] |  |  |  |  |  |  |  |  |  |  |  |  |  |  |  | -17.4 (0.9) | 7.2 (0.6) | 3 |  |  |  |  |  |  |  |  |  |  |  |  |
| **Alvinellidae** |  |  |  |  |  |  |  |  |  |  |  |  |  |  |  |  |  |  |  |  |  |  |  |  |  |  |  |  |  |  |  |  |
| *Paralvinella grasslei* | B (d, s) | [[5](#_ENREF_5)], this study |  |  |  |  |  |  |  |  |  |  |  |  |  |  |  |  |  |  |  |  |  |  |  |  | -16.7 (1.2) | -4.2 (0.8) | 6 | -18.5 (2.3) | -0.4 (0.8) | 6 |
| *Paralvinella bactericola* | B (d, s) | [[5](#_ENREF_5), [6](#_ENREF_6)], this study |  |  |  |  |  |  |  |  |  |  |  |  |  |  |  |  |  |  |  |  |  |  |  |  | -15.6 (1.3) | -0.5 (0.4) | 6 | -15.8 | -1.0 | 1 |
| **Hyalogyrinidae** | D (d) | [[7](#_ENREF_7)] |  |  |  |  |  |  |  |  |  |  |  |  |  |  |  |  |  |  |  |  |  |  |  |  |  |  |  |  |  |  |
| *Hyalogyrina sp* | D (d) | This study |  |  |  |  |  |  |  |  |  |  |  |  | -27.9 (3.4) | 8.8 (1.4) | 5 |  |  |  |  |  |  |  |  |  |  |  |  |  |  |  |
| **Bivalvia** |  |  |  |  |  |  |  |  |  |  |  |  |  |  |  |  |  |  |  |  |  |  |  |  |  |  |  |  |  |  |  |  |
| **Bathyspinulidae** |  |  |  |  |  |  |  |  |  |  |  |  |  |  |  |  |  |  |  |  |  |  |  |  |  |  |  |  |  |  |  |  |
| *Nuculana grasslei* | D (d) | [[8](#_ENREF_8)] |  |  |  | -28.8 (5.3) | 9.1 (1.6) | 7 | -35.6 (8.9) | 11.5 (0.7) | 3 | -28.5 (1.0) | 11.6 (0.1) | 3 | -31.9 (9.9) | 8.7 (1.5) | 8 | -29.8 (3.6) | 11.0 (1.8) | 2 | -20.7 (1.0) | 5.4 (2.2) | 9 |  |  |  |  |  |  |  |  |  |
| **Solemyidae** |  |  |  |  |  |  |  |  |  |  |  |  |  |  |  |  |  |  |  |  |  |  |  |  |  |  |  |  |  |  |  |  |
| *Acharax aff johnsoni* | S | [[9](#_ENREF_9)] |  |  |  | -29.7 (1.4) | -1.1 (0.7) | 4 | -28.8 (0.4) | 7.7 (0.4) | 2 |  |  |  |  |  |  |  |  |  | -30.1 (0.7) | -7.7 (0.3) | 4 |  |  |  |  |  |  |  |  |  |
| **Gastropoda** |  |  |  |  |  |  |  |  |  |  |  |  |  |  |  |  |  |  |  |  |  |  |  |  |  |  |  |  |  |  |  |  |
| **Cataegidae** | - |  |  |  |  |  |  |  |  |  |  |  |  |  |  |  |  |  |  |  |  |  |  |  |  |  |  |  |  |  |  |  |
| *Cataegis sp* | D (d) | This study, Waren pers.com. |  |  |  |  |  |  |  |  |  |  |  |  |  |  |  | -31.0 (0.4) | 8.1 (0.9) | 4 |  |  |  |  |  |  |  |  |  | -36.0 | 6.3 | 1 |
| Cataegidae juvenile | B (d) | This study |  |  |  |  |  |  |  |  |  |  |  |  |  |  |  |  |  |  |  |  |  |  |  |  |  |  |  | -17.5 | -3.0 | 1 |
| **Lepetodrilidae** | D, B (d, s) | [[7](#_ENREF_7), [10](#_ENREF_10)] |  |  |  |  |  |  |  |  |  |  |  |  |  |  |  |  |  |  |  |  |  |  |  |  |  |  |  |  |  |  |
| *Lepetodrilus guaymasensis* | B (d, s) | This study, Waren pers.com. |  |  |  |  |  |  |  |  |  |  |  |  |  |  |  | -30.0 (4.0) | 4.0 (1.5) | 15 |  |  |  |  |  |  |  |  |  |  |  |  |
| **Neolepetopsidae** |  |  |  |  |  |  |  |  |  |  |  |  |  |  |  |  |  |  |  |  |  |  |  |  |  |  |  |  |  |  |  |  |
| *Paralepetopsis sp* | D, B (d) | [[7](#_ENREF_7)], this study |  |  |  | -35.2 (4.0) | 7.3 (1.0) | 5 | -39.1 (0.8) | 9.0 (0.1) | 3 |  |  |  |  |  |  | -38.3 (1.3) | 5.5 (1.7) | 13 |  |  |  |  |  |  |  |  |  |  |  |  |
| **Neomphaloidae** | - |  |  |  |  |  |  |  |  |  |  |  |  |  |  |  |  |  |  |  |  |  |  |  |  |  |  |  |  |  |  |  |
| *Retiskenea diploura* | D (d) | This study, Waren pers.com. |  |  |  |  |  |  | -34.0 (4.8) | 11.4 (0.5) | 3 | -34.4 | 12.0 | 1 |  |  |  |  |  |  | -26.0 (0.8) | 11.3 (0.7) | 3 |  |  |  |  |  |  |  |  |  |
| **Provannidae** | D, B (d) | [[7](#_ENREF_7)] |  |  |  |  |  |  |  |  |  |  |  |  |  |  |  |  |  |  |  |  |  |  |  |  |  |  |  |  |  |  |
| *Provanna laevis* | D, B (d) | [[11](#_ENREF_11)], this study |  |  |  | -43.1 (3.7) | 8.6 (0.5) | 5 | -37.5 (1.8) | 8.8 (1.2) | 3 |  |  |  |  |  |  |  |  |  | -23.4 (2.6) | 2.8 (1.4) | 10 |  |  |  |  |  |  |  |  |  |
| *Provanna sp* | B (d) | This study, Waren pers.com. |  |  |  |  |  |  |  |  |  |  |  |  |  |  |  |  |  |  | -27.5 (4.9) | 2.6 (0.8) | 2 |  |  |  |  |  |  | -26.9 (2.9) | 0.8 (1.0) | 9 |
| **Pyramidellidae** | - |  |  |  |  |  |  |  |  |  |  |  |  |  |  |  |  |  |  |  |  |  |  |  |  |  |  |  |  |  |  |  |
| *Eulimella lomana* | D (d) | This study, Waren pers.com. |  |  |  | -33.6 (0.6) | 7.2 (0.9) | 6 | -34.1 (0.7) | 10.8 (0.3) | 6 |  |  |  |  |  |  |  |  |  |  |  |  |  |  |  |  |  |  |  |  |  |
| **Pyropeltidae** | - |  |  |  |  |  |  |  |  |  |  |  |  |  |  |  |  |  |  |  |  |  |  |  |  |  |  |  |  |  |  |  |
| *Pyropelta corymba* |  |  |  |  |  | -30.6 (1.1) | 5.1 (0.4) | 2 (10) |  |  |  |  |  |  |  |  |  |  |  |  |  |  |  |  |  |  |  |  |  |  |  |  |
| **Polychata** |  |  |  |  |  |  |  |  |  |  |  |  |  |  |  |  |  |  |  |  |  |  |  |  |  |  |  |  |  |  |  |  |
| **Ampharetidae** | D (d) | [[12](#_ENREF_12)] |  |  |  |  |  |  |  |  |  |  |  |  |  |  |  |  |  |  |  |  |  |  |  |  |  |  |  |  |  |  |
| *Eclypse cf. trilobata* | D (d) | This study | -17.4 | 18.0 | 1 |  |  |  |  |  |  |  |  |  |  |  |  |  |  |  |  |  |  |  |  |  |  |  |  |  |  |  |
| *Amphisamytha aff. fauchaldi* | D (d) | [[6](#_ENREF_6)] |  |  |  |  |  |  | -27.7 (3.3) | 10.9 (0.1) | 3 | -33.7 (0.7) | 9.1 (0.9) | 2 | -27.9 (0.8) | 10.9 (0.2) | 3 |  |  |  | -24.2 (1.3) | 7.0 (3.0) | 6 | -26.0 (0.5) | 4.7 (0.5) | 3 | -19.6 (2.2) | 0.2 (1.9) | 5 | -20.1 (2.7) | 3.4 (3.1) | 14 |
| **Archinomidae** | P, D (d) | [[12](#_ENREF_12)] |  |  |  |  |  |  |  |  |  |  |  |  |  |  |  |  |  |  |  |  |  |  |  |  |  |  |  |  |  |  |
| *Archinome rosacea* | P | [[13](#_ENREF_13)], this study |  |  |  |  |  |  |  |  |  |  |  |  |  |  |  | -30.3 (2.5) | 8.6 (2.0) | 2 | -25.6 (1.3) | 6.0 (2.0) | 9 |  |  |  |  |  |  | -14.1 (1.8) | 4.9 (2.1) | 2 |
| **Capitellidae** | D (d), C | [[12](#_ENREF_12)] |  |  |  |  |  |  |  |  |  |  |  |  |  |  |  |  |  |  |  |  |  |  |  |  |  |  |  |  |  |  |
| *Barantolla sp* | D (d) | [[12](#_ENREF_12)], this study | -17.4 | 18.1 | 1 |  |  |  |  |  |  |  |  |  |  |  |  |  |  |  |  |  |  |  |  |  |  |  |  |  |  |  |
| **Cirratulidae** | D (d, s) | [[12](#_ENREF_12)] |  |  |  |  |  |  |  |  |  |  |  |  |  |  |  |  |  |  |  |  |  |  |  |  |  |  |  |  |  |  |
| *Cirratulus sp* | D (d, s) | This study | -17.6 (0.4) | 17.1 (0.7) | 3 | -28.3 (1.5) | 10.9 (2.0) | 10 | -24.1 (1.3) | 15.3 (1.5) | 6 |  |  |  |  |  |  | -23.7 (3.0) | 17.1 (0.3) | 2 |  |  |  |  |  |  |  |  |  |  |  |  |
| *Aphelochaeta sp* | D (d, s) | This study |  |  |  |  |  |  |  |  |  |  |  |  | -39.2 (2.6) | 11.1 (1.8) | 7 |  |  |  | -20.1 | 15.5 | 1 |  |  |  |  |  |  |  |  |  |
| **Cossuridae** | D (d) | [[12](#_ENREF_12)] |  |  |  |  |  |  |  |  |  |  |  |  |  |  |  |  |  |  |  |  |  |  |  |  |  |  |  |  |  |  |
| *Cossura sp* | D (d) | This study |  |  |  |  |  |  | -47.0 | 13.3 | 1 |  |  |  |  |  |  |  |  |  |  |  |  |  |  |  |  |  |  |  |  |  |
| **Dorvilleidae** | P, D (d), B (d), C | [[12](#_ENREF_12), [14](#_ENREF_14), [15](#_ENREF_15)] |  |  |  |  |  |  |  |  |  |  |  |  |  |  |  |  |  |  |  |  |  |  |  |  |  |  |  |  |  |  |
| Dorvilleidae Unind | B (d) | This study |  |  |  |  |  |  |  |  |  |  |  |  |  |  |  | -32.9 | 6.4 | 1 (10) |  |  |  |  |  |  |  |  |  |  |  |  |
| *Parougia sp* | B (d) | This study |  |  |  |  |  |  | -77.1 (2.4) | 8.2 (0.8) | 4 |  |  |  | -78.0 (0.4) | 8.2 (1.0) | 3 |  |  |  |  |  |  |  |  |  |  |  |  |  |  |  |
| *Ophryotrocha platykephale* | D (d), B (d) | This study |  |  |  |  |  |  |  |  |  | -25.4 | 2.8 | 1 (10) | -21.9 (1.6) | 11.1 (0.1) | 3 |  |  |  |  |  |  | -29.2 (3.8) | -0.1 (1.1) | 2 |  |  |  |  |  |  |
| *Ophryotrocha akessoni* | P, D (d), B (d) | This study |  |  |  |  |  |  |  |  |  |  |  |  |  |  |  |  |  |  |  |  |  | -26.6 | 1.1 | 1 | -16.6 (1.1) | -3.2 (2.4) | 5 | -17.5 (1.7) | 1.4 (1.2) | 6 |
| *Exallopus jumarsi* | B (d) | This study |  |  |  |  |  |  |  |  |  |  |  |  |  |  |  |  |  |  | -24.0 (2.7) | 0.0 (1.3) | 9 |  |  |  |  |  |  |  |  |  |
| **Flabelligeridae** | D (d) | [[12](#_ENREF_12)] |  |  |  |  |  |  | -31.3 | 11.2 | 1 |  |  |  |  |  |  |  |  |  |  |  |  |  |  |  |  |  |  |  |  |  |
| **Glyceridae** | P | [[12](#_ENREF_12)] |  |  |  |  |  |  |  |  |  |  |  |  |  |  |  |  |  |  |  |  |  |  |  |  |  |  |  |  |  |  |
| *Glycera sp* | P | This study |  |  |  |  |  |  | -22.4 | 19.5 | 1 |  |  |  |  |  |  |  |  |  |  |  |  |  |  |  |  |  |  |  |  |  |
| **Hesionidae** | P, B (d), D (d) | [[12](#_ENREF_12)] |  |  |  |  |  |  |  |  |  |  |  |  |  |  |  |  |  |  |  |  |  |  |  |  |  |  |  |  |  |  |
| *Sirsoe grasslei* | D (d), B (d) | This study |  |  |  |  |  |  | -28.7 | 11.2 | 1 | -24.3 | 1.5 | 1 |  |  |  |  |  |  |  |  |  |  |  |  |  |  |  |  |  |  |
| **Lacydonidae** | P, D (d) | [[12](#_ENREF_12)] |  |  |  |  |  |  |  |  |  |  |  |  |  |  |  |  |  |  |  |  |  |  |  |  |  |  |  |  |  |  |
| *Lacydonia sp* | D (d) | This study |  |  |  |  |  |  | -32.9 (1.1) | 12.0 (1.2) | 2 |  |  |  |  |  |  |  |  |  |  |  |  |  |  |  |  |  |  |  |  |  |
| **Lumbrineridae** | P, D (d) | [[12](#_ENREF_12)] |  |  |  |  |  |  |  |  |  |  |  |  |  |  |  |  |  |  |  |  |  |  |  |  |  |  |  |  |  |  |
| *Lumbrineris sp* | P | This study |  |  |  |  |  |  | -22.2 | 17.1 | 1 |  |  |  |  |  |  |  |  |  |  |  |  |  |  |  |  |  |  |  |  |  |
| **Maldanidae** | D (d, s), B (d, s) | [[12](#_ENREF_12)] |  |  |  |  |  |  |  |  |  |  |  |  |  |  |  |  |  |  |  |  |  |  |  |  |  |  |  |  |  |  |
| *Nicomache venticola* | B (d, s) | This study |  |  |  | -46.9 (9.1) | 7.9 (1.8) | 12 | -37.3 (5.3) | 9.6 (1.2) | 2 |  |  |  |  |  |  | -34.1 | 12.6 | 1 | -25.7 | 8.5 | 1 |  |  |  |  |  |  | -31.2 (1.9) | 8.7 (0.8) | 2 |
| *Notoproctus sp* | D (d, s) | This study |  |  |  | -19.1 (0.8) | 6.3 (0.6) | 2 |  |  |  |  |  |  |  |  |  |  |  |  |  |  |  |  |  |  |  |  |  |  |  |  |
| **Nautiniellidae** | C | [[16-18](#_ENREF_16)], this study |  |  |  | -35.0 | 7.3 | 1 | -28.3 | 9.8 | 1 |  |  |  |  |  |  |  |  |  |  |  |  |  |  |  |  |  |  |  |  |  |
| **Nereididae** | P, D (d) | [[12](#_ENREF_12)] |  |  |  |  |  |  |  |  |  |  |  |  |  |  |  |  |  |  |  |  |  |  |  |  |  |  |  |  |  |  |
| Nereididae Unind | D (d)? | This study | -17.1 (0.3) | 15.3 (1.5) | 2 |  |  |  |  |  |  |  |  |  |  |  |  |  |  |  |  |  |  |  |  |  |  |  |  |  |  |  |
| *Nereis sandersi* | P, D (d) | This study |  |  |  | -44.1 (2.0) | 6.0 (0.1) | 2 | -37.0 (9.1) | 13.5 (0.7) | 3 |  |  |  | -36.1 (3.5) | 10.2 (2.7) | 2 | -40.1 | 11.4 | 1 | -24.2 (2.0) | 7.7 (1.9) | 3 |  |  |  | -34.6 | 6.9 | 1 | -30.2 (4.8) | 6.1 (1.7) | 3 |
| **Paraonidae** | D (d) | [[12](#_ENREF_12)] |  |  |  |  |  |  |  |  |  |  |  |  |  |  |  |  |  |  |  |  |  |  |  |  |  |  |  |  |  |  |
| *Levinsenia sp* | D (d) | This study | -17.6 | 19.5 | 1(3) |  |  |  | -23.2 | 16.5 | 1 |  |  |  |  |  |  |  |  |  |  |  |  |  |  |  |  |  |  |  |  |  |
| **Phyllodocidae** | P, D (d), C | [[12](#_ENREF_12)] |  |  |  |  |  |  |  |  |  |  |  |  |  |  |  | -19.2 (0.1) | 7.3 (2.7) | 4 |  |  |  |  |  |  |  |  |  |  |  |  |
| Phyllodocidae unind | C | This study |  |  |  |  |  |  |  |  |  |  |  |  |  |  |  |  |  |  |  |  |  |  |  |  |  |  |  |  |  |  |
| **Pilargidae** | P | [[12](#_ENREF_12)] |  |  |  |  |  |  |  |  |  |  |  |  |  |  |  |  |  |  |  |  |  |  |  |  |  |  |  |  |  |  |
| *Sigambra* sp | P | This study | -21.8 | 15.4 | 1 |  |  |  |  |  |  |  |  |  |  |  |  |  |  |  | -24.1 | 12.3 | 1 |  |  |  |  |  |  |  |  |  |
| **Polynoidae** | P, B (d), D (d), C | [[12](#_ENREF_12)] |  |  |  |  |  |  |  |  |  |  |  |  |  |  |  |  |  |  |  |  |  |  |  |  |  |  |  |  |  |  |
| Polynoidae unind | P | This study | -16.4 (1.6) | 21.3 (0.9) | 4 |  |  |  |  |  |  |  |  |  |  |  |  |  |  |  |  |  |  |  |  |  |  |  |  |  |  |  |
| *Bathykurila guaymasensis* | P, B (d), D (d) | [[19](#_ENREF_19)], this study |  |  |  | -30.4 | 0.9 | 1 | -27.8 (0.1) | 12.6 (1.8) | 3 | -27.9 (0.5) | 11.4 (0.8) | 4 | -27.5 (1.3) | 10.8 (1.0) | 4 |  |  |  | -23.3 (1.5) | 8.0 (0.6) | 3 |  |  |  | -9.6 (1.7) | -2.3 (2.5) | 2 |  |  |  |
| *Branchinotogluma sandersi* | P, B (d), D (d), C | [[6](#_ENREF_6), [20](#_ENREF_20)], this study |  |  |  |  |  |  |  |  |  | -33.3 | 10.5 | 1 |  |  |  | -19.0 (1.4) | 3.4 (0.6) | 5 |  |  |  |  |  |  | -17.7 (1.0) | -0.6 (0.8) | 7 | -23.7 (2.7) | 1.1 (1.3) | 6 |
| *Branchinotogluma hessleri* | P | This study |  |  |  |  |  |  |  |  |  |  |  |  | -32.7 (5.5) | 14.3 (1.2) | 4 |  |  |  |  |  |  |  |  |  |  |  |  |  |  |  |
| *Branchiplicatus cupreus* | - |  |  |  |  |  |  |  |  |  |  |  |  |  |  |  |  |  |  |  |  |  |  |  |  |  | -33.9 | 1.4 | 1 |  |  |  |
| *Lepidonotopodium riftense* | P, B (d) | [[20](#_ENREF_20)], this study |  |  |  |  |  |  |  |  |  |  |  |  |  |  |  |  |  |  |  |  |  |  |  |  |  |  |  | -14.9 (1.7) | 3.3 (2.0) | 4 |
| **Serpulidae** | D (s), B (s) | [[12](#_ENREF_12), [21](#_ENREF_21)] |  |  |  |  |  |  |  |  |  |  |  |  |  |  |  | -42.4 (1.7) | 5.8 (1.0) | 9 |  |  |  |  |  |  |  |  |  |  |  |  |
| Serpulidae unind | B (s) | This study |  |  |  |  |  |  |  |  |  |  |  |  |  |  |  |  |  |  |  |  |  |  |  |  |  |  |  |  |  |  |
| **Sigalionidae** | P | [[12](#_ENREF_12)] |  |  |  |  |  |  |  |  |  |  |  |  |  |  |  |  |  |  |  |  |  |  |  |  |  |  |  |  |  |  |
| *Neoleanira sp* | D (d)? | This study | -17.6 (0.0) | 16.4 (4.8) | 2 |  |  |  |  |  |  |  |  |  |  |  |  |  |  |  |  |  |  |  |  |  |  |  |  |  |  |  |
| **Spionidae** | D (d, s) | [[12](#_ENREF_12)] |  |  |  |  |  |  |  |  |  |  |  |  |  |  |  |  |  |  |  |  |  |  |  |  |  |  |  |  |  |  |
| Spionidae unind | D (d, s) | This study |  |  |  |  |  |  |  |  |  |  |  |  |  |  |  | -35.7 | 10.0 | 1 (3) |  |  |  |  |  |  | -36.3 (1.2) | 1.7 (0.5) | 3 |  |  |  |
| *Spiophanes sp* | D (d, s) | This study | -17.5 | 15.9 | 1 |  |  |  |  |  |  |  |  |  |  |  |  |  |  |  |  |  |  |  |  |  |  |  |  |  |  |  |
| *Lindaspio dibranchiata* | B (d, s) | This study |  |  |  |  |  |  |  |  |  |  |  |  |  |  |  |  |  |  | -18.8 | -2.8 | 1 |  |  |  |  |  |  |  |  |  |
| **Terrebellidae** | D (d, s) | [[12](#_ENREF_12)] |  |  |  |  |  |  |  |  |  |  |  |  |  |  |  |  |  |  |  |  |  |  |  |  |  |  |  |  |  |  |
| Terrebellidae | D (d, s) | This study |  |  |  | -35.9 (3.2) | 10.4 (1.7) | 9 | -36.1 | 12.3 | 1 |  |  |  | -28.3 (0.7) | 12.7 (2.2) | 5 | -29.4 (3.1) | 10.9 (1.8) | 7 | -25.0 (1.4) | 11.0 (2.5) | 3 |  |  |  |  |  |  | -30.9 (3.2) | 8.9 (0.8) | 3 |
| **Others** |  |  |  |  |  |  |  |  |  |  |  |  |  |  |  |  |  |  |  |  |  |  |  |  |  |  |  |  |  |  |  |  |
| **Actinaria** | D (s) | [[22](#_ENREF_22), [23](#_ENREF_23)] |  |  |  |  |  |  |  |  |  |  |  |  |  |  |  |  |  |  |  |  |  |  |  |  |  |  |  |  |  |  |
| Actinaria unind | D (s), B (s) | This study |  |  |  |  |  |  | -30.4 (1.4) | 12.2 (0.4) | 3 |  |  |  | -29.9 (1.2) | 6.9 (0.2) | 3 |  |  |  |  |  |  |  |  |  |  |  |  | -24.4 (1.6) | 12.1 (1.1) | 2 |
| **Amphipoda** | P, D (d) | [[24](#_ENREF_24), [25](#_ENREF_25)] |  |  |  |  |  |  |  |  |  |  |  |  |  |  |  |  |  |  |  |  |  |  |  |  |  |  |  |  |  |  |
| Amphipoda unind. | D (d) | This study |  |  |  |  |  |  |  |  |  |  |  |  |  |  |  |  |  |  | -27.1 (0.4) | 10.0 (1.5) | 2 |  |  |  | -18.1 | 17.2 | 1 | -22.5 | 11.4 | 1 |
| **Aplacophora** | P, D (d) | [[26](#_ENREF_26)] |  |  |  |  |  |  |  |  |  |  |  |  |  |  |  |  |  |  |  |  |  |  |  |  |  |  |  |  |  |  |
| Aplacophora unind | D (d) | This study | -17.0 | 15.6 | 1 |  |  |  | -20.7 (0.8) | 15.3 (1.3) | 3 |  |  |  |  |  |  |  |  |  | -35.7 | 8.8 | 1 |  |  |  |  |  |  |  |  |  |
| **Nemertina** | P, D (d) | [[22](#_ENREF_22), [24](#_ENREF_24), [26](#_ENREF_26)] |  |  |  |  |  |  |  |  |  |  |  |  |  |  |  |  |  |  |  |  |  |  |  |  |  |  |  |  |  |  |
| Nemertina unind | P | This study | -16.4 | 21.5 | 1 |  |  |  | -34.3 (0.1) | 12.8 (1.2) | 2 |  |  |  |  |  |  |  |  |  |  |  |  |  |  |  |  |  |  |  |  |  |
| **Ophiuridae** | D (d) | [[24](#_ENREF_24)] |  |  |  |  |  |  |  |  |  |  |  |  |  |  |  |  |  |  |  |  |  |  |  |  |  |  |  |  |  |  |
| *Ophiura sp* | D (d), B (d) | This study |  |  |  | -30.6 (1.3) | 2.1 (2.0) | 15 | -28.5 (0.6) | 11.1 (0.6) | 11 | -28.3 | 11.1 | 1 | -30.9 (2.5) | 6.3 (3.1) | 9 |  |  |  |  |  |  |  |  |  |  |  |  |  |  |  |
| **Munidopsidae** |  |  |  |  |  |  |  |  |  |  |  |  |  |  |  |  |  |  |  |  |  |  |  |  |  |  |  |  |  |  |  |  |
| *Munidopsis alvisca* | D (d), B (d) | [[27](#_ENREF_27)], this study |  |  |  |  |  |  | -33.4 | 12.1 | 1 |  |  |  |  |  |  | -38.6 (2.2) | 10.9 (0.9) | 3 | -22.1 (1.1) | 3.1 (0.7) | 6 |  |  |  |  |  |  | -31.2 (4.3) | 6.4 (1.6) | 3 |
| *Munidopsis diomedae* | D (d) | [[27](#_ENREF_27)] | -15.9 (0.3) | 21.2 (0.7) | 3 |  |  |  |  |  |  |  |  |  |  |  |  |  |  |  |  |  |  |  |  |  |  |  |  |  |  |  |
| **Zoarcidae** | P, D (d) | [[26](#_ENREF_26)] |  |  |  |  |  |  |  |  |  |  |  |  |  |  |  |  |  |  |  |  |  |  |  |  |  |  |  |  |  |  |
| Zoarcidae unind | P | This study | -15.6 (0.9) | 19.1 (2.2) | 2 |  |  |  |  |  |  |  |  |  |  |  |  |  |  |  |  |  |  |  |  |  |  |  |  |  |  |  |
| **Copepoda** | D (d), B (d), C | [[26](#_ENREF_26)] |  |  |  |  |  |  |  |  |  |  |  |  |  |  |  |  |  |  |  |  |  |  |  |  |  |  |  |  |  |  |
| *Vurdolaker acharaxis sp nov* | C | This study |  |  |  |  |  |  | -27.9 (0.2) | 9.9 (0.9) | 4 (2) |  |  |  |  |  |  |  |  |  |  |  |  |  |  |  |  |  |  |  |  |  |
| *Aphotopontius mammillatus* | B (d), D (d) | This study |  |  |  |  |  |  |  |  |  |  |  |  |  |  |  |  |  |  |  |  |  |  |  |  | -20.8 (0.0) | -1.9 (0.0) | 2 (100) | -19.7 (0.7) | -2.2 (0.2) | 2 (100) |
| *Stygiopontius flexus* | D (d) | This study |  |  |  |  |  |  |  |  |  |  |  |  |  |  |  |  |  |  |  |  |  |  |  |  | -15.3 (0.2) | -4.0 (0.1) | 2(100) |  |  |  |
| **Nematoda** | D (d) | [[26](#_ENREF_26)] |  |  |  |  |  |  |  |  |  |  |  |  |  |  |  |  |  |  |  |  |  |  |  |  |  |  |  |  |  |  |
| Desmodoridae | - |  |  |  |  |  |  |  |  |  |  |  |  |  |  |  |  |  |  |  |  |  |  |  |  |  | -34.8 (0.1) | 2.2 (0.2) | 2 | -29.9 | 4.2 | 1 |
| **Total mean** |  |  | -17.1 (1.4) | 18.4 (2.7) |  | -34.8 (7.4) | 5.9 (4.1) |  | -31.0 (5.9) | 11.7 (2.5) |  | -29.3 (3.2) | 9.7 (3.4) |  | -31.0 (6.0) | 9.8 (2.9) |  | -31.1 (8.1) | 6.7 (3.5) |  | -25.6 (4.7) | 3.7 (5.1) |  | -27.2 (2.4) | 2.5 (2.5) |  | -16.9 (2.6) | -1.8 (2.1) |  | -17.7 (3.5) | 1.5 (2.6) |  |
| **Primary consumer’s mean** |  |  | -17.1 | 12.0 |  | -32.8 | 1.1 |  | -39.0 | 8.8 |  | -24.8 | 2.2 |  | -46.3 | 7.1 |  | -27.0 | 5.2 |  | -26.9 | -3.1 |  | -27.9 | 0.5 |  | -15.8 | -2.7 |  | -16.9 | -0.7 |  |
| **Secondary consumer ‘s mean** |  |  | -17.4 | 17.6 |  | -35.0 | 8.1 |  | -30.7 | 13.1 |  | -31.0 | 11.0 |  | -30.4 | 11.0 |  | -32.5 | 11.2 |  | -25.0 | 8.0 |  | -26.0 | 4.7 |  | -17.6 | -0.3 |  | -17.5 | 3.4 |  |
| **Local MOP mean** |  |  | -20.5 (0.1) | 8.6 (0.7) | 3 | -22.8 (0.4) | 4.4 (0.2) | 3 | -24.9 (1.3) | 4.7 (1.7) | 3 | -24.4 (0.7) | 8.6 (0.6) | 3 | -24.9 (1.4) | 9.0 (1.3) | 3 | -22.8 (0.2) | 9.6 (0.2) | 3 | -21.2 (0.6) | 8.2 (0.8) | 3 | -22.4 (0.7) | 4.7 (0.9) | 3 |  |  |  |  |  |  |
| **Microbial mat** |  |  |  |  |  |  |  |  |  |  |  | -26.4 | 3.5 | 1 |  |  |  |  |  |  |  |  |  |  |  |  | -29.8 | 1.6 | 1 |  |  |  |
| **Methane** |  |  |  |  |  | -53.0 |  | 1 | -51.0 |  | 1 | -53.0 |  | 1 |  |  |  |  |  |  |  |  |  | -41.1 |  | 1 | -41.6 |  | 1 | -42.6 |  | 1 |
| **Methane mean** |  |  | -52.3 (1.2) | | | | | | | | | | | | | | | | | | -41.8 (0.8) | | | | | | | | | | | |
| **Macrofaunal density (ind.m^-2^)** |  |  | 569 (328) |  |  | 1426  (903) | |  | 11,037  (3090) | |  | 880  (762) | |  | 24,889 | |  | 8028.0 | |  | 1667  (735) | |  | 708  (20) | |  | 2614.0 | |  | 94,348 | |  |
| **Macrofaunal alpha diversity (S_41_)** |  |  | 14.0 |  |  | 11.1 | |  | 12.2 | |  | 6.4 | |  | 2.1 | |  | 10.9 | |  | 10.3 | |  | 5.4 | |  | 3.0 | |  | 2.0 | |  |

1. Scott KM, Fisher CR. Physiological ecology of sulfide metabolism in hydrothermal vent and cold seep vesicomyid clams and vestimentiferan tube worms. Am Zool. 1995;35(2):102-11. PubMed PMID: WOS:A1995RH78900004.

2. Grassle J, Brown-Leger L, Morse-Porteous L, Petrecca R, Williams I. Deep-sea fauna of sediments in the vicinity of hydrothermal vents. Bull Biol Soc Wash. 1985;(6):443-52.

3. Fisher C. Chemoautotrophic and methanotrophic symbioses in marine-invertebrates. Rev Aquat Sci. 1990;2(3-4):399-436.

4. Cavanaugh C. Symbiosis of chemoautotrophic bacteria and marine invertebrates from hydrothermal vents and reducing sediments. Bull Biol Soc Wash. 1985;6:373-88.

5. Desbruyères D, Laubier L. Systematics, phylogeny, ecology and distribution of the Alvinellidae (Polychaeta) from deep-sea hydrothermal vents. Ophelia. 1991:31-45. PubMed PMID: WOS:A1991FP60800003.

6. Soto LA. Stable carbon and nitrogen isotopic signatures of fauna associated with the deep-sea hydrothermal vent system of Guaymas Basin, Gulf of California. Deep Sea Res Part II Top Stud Oceanogr. 2009;56(19):1675-82.

7. Warén A, Bouchet P. New gastropods from deep-sea hydrocarbon seeps off West Africa. Deep Sea Res Part II Top Stud Oceanogr. 2009;56(23):2326-49. doi: 10.1016/j.dsr2.2009.04.013.

8. Allen J. A new deep-water hydrothermal species of Nuculana (Bivalvia: Protobranchia) from the Guaymas Basin. Malacologia. 1993;35(1):141-51.

9. Barry JP, Buck K, Goffredi SK, Hashimoto J. Ultrastructure studies of two chemosynthetic invertebrate–bacterial symbioses (Lamellibrachia sp. and Acharax sp.) from the Hatsushima cold seeps in Sagami Bay, Japan. Jamstec J Deep Sea Res. 2000;16:91-9.

10. Bates AE. Feeding strategy, morphological specialisation and presence of bacterial episymbionts in lepetodrilid gastropods from hydrothermal vents. Mar Ecol Prog Ser. 2007;347:87-99. doi: 10.3354/meps07020. PubMed PMID: WOS:000250978500008.

11. Warén A, Ponder WF. New species, anatomy, and systematic position of the hydrothermal vent and hydrocarbon seep gastropod family Provannidae fam. n. (Caenogastropoda). Zool Scr. 1991;20(1):27-56.

12. Jumars PA, Dorgan KM, Lindsay SM. Diet of Worms Emended: An Update of Polychaete Feeding Guilds. Ann Rev Mar Sci. 2015;7:497-520. doi: doi:10.1146/annurev-marine-010814-020007. PubMed PMID: 25251269.

13. Ward ME, Jenkins CD, Van Dover CL. Functional morphology and feeding strategy of the hydrothermal-vent polychaete Archinome rosacea (family Archinomidae). Can J Zool. 2003;81(4):582-90. doi: Doi 10.1139/Z03-034. PubMed PMID: WOS:000183101400003.

14. Levin LA, Ziebis W, F. Mendoza G, Bertics VJ, Washington T, Gonzalez J, et al. Ecological release and niche partitioning under stress: Lessons from dorvilleid polychaetes in sulfidic sediments at methane seeps. Deep Sea Res Part II Top Stud Oceanogr. 2013;92(0):214-33. doi: <http://dx.doi.org/10.1016/j.dsr2.2013.02.006>.

15. Thurber AR, Levin LA, Orphan VJ, Marlow JJ. Archaea in metazoan diets: implications for food webs and biogeochemical cycling. ISME J. 2012;6(8):1602-12. doi: <http://www.nature.com/ismej/journal/v6/n8/suppinfo/ismej201216s1.html>.

16. Van Dover C, Aharon P, Bernhard J, Caylor E, Doerries M, Flickinger W, et al. Blake Ridge methane seeps: characterization of a soft-sediment, chemosynthetically based ecosystem. Deep Sea Res Part I Oceanogr Res Pap. 2003;50(2):281-300.

17. Becker EL, Cordes EE, Macko SA, Lee RW, Fisher CR. Using Stable Isotope Compositions of Animal Tissues to Infer Trophic Interactions in Gulf of Mexico Lower Slope Seep Communities. PloS one. 2013;8(12):e74459.

18. Ravara A, Cunha MR, Rodrigues CF. The occurrence of Natsushima bifurcata (Polychaeta: Nautiliniellidae) in Acharax hosts from mud volcanoes in the Gulf of Cadiz (south Iberian and north Moroccan Margins). Sci Mar. 2007;71(1):95-100.

19. Glover AG, Goetze E, Dahlgren TG, Smith CR. Morphology, reproductive biology and genetic structure of the whale‐fall and hydrothermal vent specialist, *Bathykurila guaymasensis* Pettibone, 1989 (Annelida: Polynoidae). Mar Ecol. 2005;26(3‐4):223-34.

20. Gaudron SM, Lefebvre S, Nunes Jorge A, Gaill F, Pradillon F. Spatial and temporal variations in food web structure from newly-opened habitat at hydrothermal vents. Mar Environ Res. 2012;77:129-40. doi: 10.1016/j.marenvres.2012.03.005.

21. Levin LA, Orphan VJ, Rouse GW, Rathburn AE, Ussler W, Cook GS, et al. A hydrothermal seep on the Costa Rica margin: middle ground in a continuum of reducing ecosystems. Proceedings of the Royal Society B: Biological Sciences. 2012;279(1738):2580-8. doi: 10.1098/rspb.2012.0205.

22. Moreno RA, Sepúlveda RD, Badano EI, Thatje S, Rozbaczylo N, Carrasco FD. Subtidal macrozoobenthos communities from northern Chile during and post El Nino 1997–1998. Helgol Mar Res. 2008;62(1):45-55.

23. Orejas Saco del Valle C. Role of benthic cnidarians in energy transfer processes in the Southern Ocean marine ecosystem (Antarctica). Bremerhaven: Alfred Wegener Institute for Polar and Marine Research; 2001. 186 p.

24. Long B, Poiner I. Infaunal benthic community structure and function in the Gulf of Carpentaria, northern Australia. Mar Freshwater Res. 1994;45(3):293-316.

25. Thiel M, González E, Balanda M-J, Haye P, Heard R, Watling L. Diversity of Chilean peracarids (Crustacea: Malacostraca). In: Hendrickx ME, editor. Contributions to the study of East-Pacific crustaceans. México: Instituto de Ciencias del Mar y Limnología; 2004. p. 177-89.

26. Bergquist DC, Eckner JT, Urcuyo IA, Cordes EE, Hourdez S, Macko SA, et al. Using stable isotopes and quantitative community characteristics to determine a local hydrothermal vent food web. Mar Ecol Prog Ser. 2007;330:49-65.

27. Escobar-Briones E, Morales P, Cienfuegos E, González M. Carbon sources and trophic position of two abyssal species of Anomura, Munidopsis alvisca (Galatheidae) and Neolithodes diomedeae (Lithodidae). Contributions to the study of East Pacific Crustaceans. 2002;1:37-43.
