# Supplementary material for: Food-Web Complexity in Guaymas Basin Hydrothermal Vents and Cold Seeps
Source: PLoS One. 2016 Sep 28;11(9):e0162263. doi: 10.1371/journal.pone.0162263 (PMC5040445; doi:10.1371/journal.pone.0162263)
Supplement: S1 Fig — (DOCX) [file pone.0162263.s002.docx]

**S1 Fig. Illustration of the copepod species nov deeply attached to *Acharax* aff. *johnsoni* gill lamellae (A) and zoom on the copepod head (B).**

| A  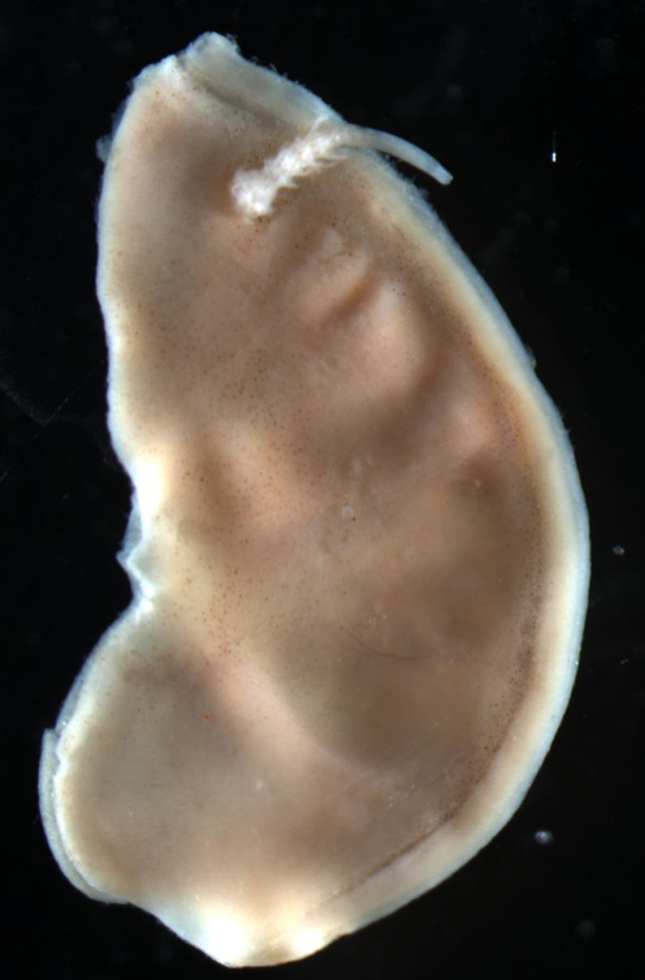 | B  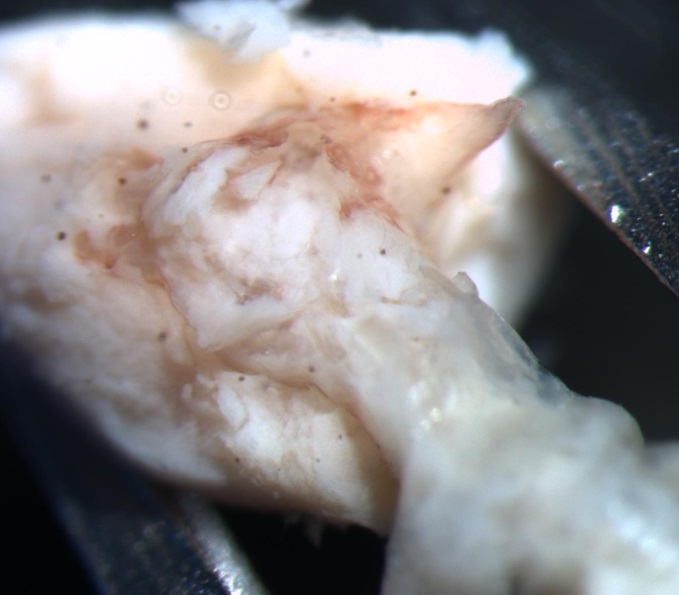 |
| --- | --- |
